# A novel method for standardised imaging of corneal subbasal nerves by in vivo confocal microscopy – a pilot validation study

**DOI:** 10.1038/s41598-026-54268-8

**Published:** 2026-06-09

**Authors:** Siv A. Sandvik, Eilin Lundanes, Stephan Allgeier, Emanuele Käser, Neil Lagali, Jorunn Lid, Tove Lise Morisbakk, Vibeke Sundling

**Affiliations:** 1 National Centre for Optics, Vision and Eye Care, Department of Optometry, Radiography and Lighting Design, University of South-Eastern Norway, Postbox 4, Borre, 3199 Norway; 2 Institute for Automation and Applied Informatics, Karlsruhe Institute of Technology (KIT), Postfach 3640, 76021 Karlsruhe, Germany; 3 Institute of Optometry, University of Applied Sciences and Arts Northwestern Switzerland FHNW, Olten, 4600 Switzerland; 4 Department of Biomedical Engineering, University of Basel, Basel, Switzerland; 5 Department of Biomedical and Clinical Sciences, Linköping University, Linköping, SE-581 83 Sweden

**Keywords:** Imaging method, In vivo confocal microscopy, Cornea, Nerve fibres, Inferior whorl, Computational biology and bioinformatics, Diseases, Medical research

## Abstract

In vivo confocal microscopy is a valuable tool for evaluating corneal subbasal nerve plexus (SBNP). However, lack of standardised, repeatable, and reliable methods is a challenge, particularly in clinical, multi-centre, and longitudinal settings. To address this, we developed a standardised imaging method using the inferior whorl (IW) region of the SBNP as a reference for imaging a predefined 1.5 mm^2^ central region of interest (ROI). No systematic differences were observed either between the two operators or between test–retest assessments performed by the same operator. A full 400 μm diameter circle around the IW was imaged in 85% of the mosaics. The inter-operator repeatability for the x- and the y-coordinates when defining the centre of the IW was 0.996 (95% CI [0.991, 0.999]) and 0.997 (95% CI [0.994, 0.999]), respectively. For 61% of the mosaics, the ROI was accurately positioned and fully imaged. By anchoring the ROI to the IW, the approach enables more repeatable and anatomically consistent imaging, supporting improved comparability across examinations. While the method shows promise, further refinement and technical development are required to enhance its reliability and robustness.

**Supplementary Information:**

The online version contains supplementary material available at 10.1038/s41598-026-54268-8.

## Introduction

Corneal in vivo confocal microscopy (IVCM) is a fast, non-invasive imaging method that enables high quality images of corneal structures to be obtained, spanning the epithelium to the endothelium^1^. In particular, IVCM has been applied to evaluate the corneal nerve fibres in individuals with various conditions, which involve nerve alterations or neurodegeneration, such as diabetes and Parkinson’s disease^1–7^. Compared to skin biopsy, IVCM offers the potential of a rapid and less invasive evaluation of the peripheral, small nerve fibres^8^. Nevertheless, several challenges must be addressed before the instrument can be widely adopted and utilized in clinical practice. This includes standardised protocols and guidelines to ensure consistent and reliable imaging, adequate training for operators, systems for managing and analysing the data, clinical validation, and cost and accessibility.

Despite publication of protocols aiming to standardise image acquisition and reduce biases, a robust, repeatable image acquisition protocol is yet to be developed^9–14^. Currently, there is no gold standard or consensus for IVCM image acquisition^12,15^. A meta-analysis of corneal confocal microscopy assessing diabetic peripheral neuropathy highlighted that the wide variation of image acquisition and analysis methods makes comparison across studies difficult^16^. Similarly, a recent scoping review of corneal nerve fibre evaluation in diabetes emphasised that methodology heterogeneity and lack of standardised reporting standards hinder reliable study replications^17^.

Challenges in image acquisition include operator expertise, the uneven distribution of nerve fibres across the cornea, and the small field of view of the instrument^18,19^. Image quality, and therefore corneal nerve fibre quantification, can be biased by the level of operator expertise^20,21^. Further, the field of view of one single IVCM image represents 400 μm x 400 μm covering less than 1% of the central cornea^22^. Hence, multiple images are needed to account for the variability in the nerve fibre distribution. In published studies of corneal nerves in diabetes, three to six images from each eye are frequently used for nerve fibre quantification^17^. Various adaptations have been proposed to account for the inhomogeneity of corneal nerve fibre distribution^23,24^, ranging from five single images to mosaic creation of a 1.5 mm^2^ area or even larger^25^. To ensure more consistent and robust image acquisition, different approaches have been suggested. Some involve the use of an extended fixation target^10,13,26^ while others created wide-field mosaics from multiple single images^9,11^. Additionally, a method has been described to combine the use of a fixation target with the application of the inferior whorl (IW) as a reference point to collect single images at 30 specified positions^13^.

For IVCM to become a routine instrument for imaging corneal nerve fibres in a clinical setting, the methodology should be standardised, rapid, repeatable, reliable and robust. The aim of this pilot validation study was to explore a novel method for standardised in vivo confocal imaging of the corneal subbasal nerve plexus (SBNP). The method comprises imaging, mosaicking, and identification of a region of interest (ROI). Both image acquisition and ROI delineation are standardised using the IW as an anatomical reference landmark.

## Methods

### Proposed imaging method

#### Theoretical considerations

A region of interest (ROI) was defined to include the corneal apex and cover a total area of 1.5 mm^2^ of the corneal subbasal nerve plexus (SBNP). The size and placement of the ROI were based on three considerations, addressing both qualitative and quantitative aspects of the SBNP.

First, we defined the ROI to be larger than ROIs typically used in previous studies to more effectively account for the well-known inhomogeneous distribution of the corneal nerve fibres. A larger sampling area is expected to enhance the robustness of quantitative metrics and reduce selection bias^23,24^. Second, the IW represent a unique structural landmark characterized by the convergence of nerve fibres into a whorl or a seam-like configuration^18,27,28^. This distinctive pattern serves as a reliable anatomical reference point, allowing the same region to be consistently re-located in subsequent examinations. Third, subbasal nerve fibres in the central cornea are predominantly vertically oriented along the superior-inferior corneal axis^29^. This characteristic pattern provides an additional internal check of positional accuracy, as the expected nerve orientation within the ROI can be used to confirm that the imaging is indeed centered on the correct anatomical region.

**Fig. 1:**
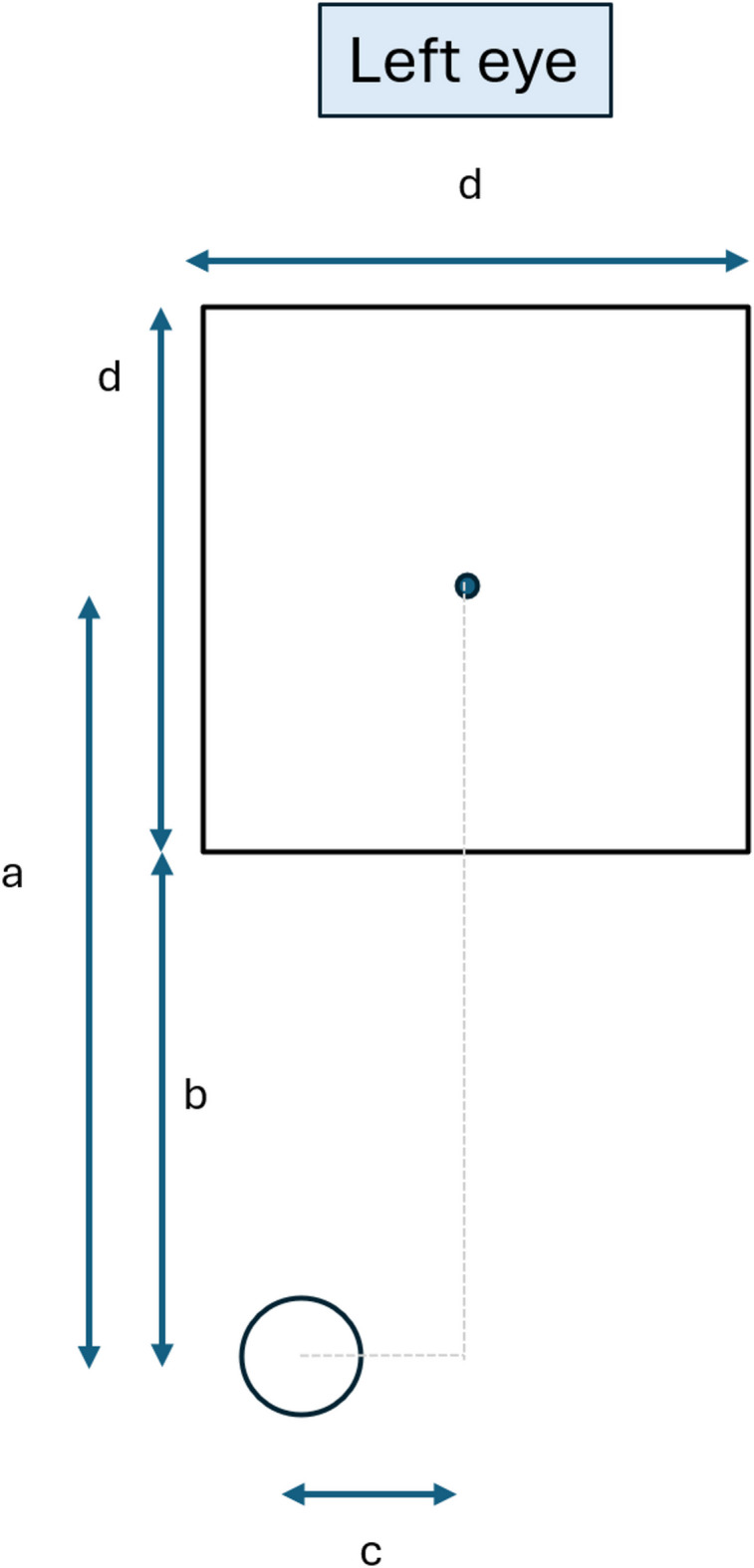
The position of the region of interest (ROI) in relation to the inferior whorl (IW) in the left eye; a = The distance from the centre of IW to a theoretical corneal apex: 1.6125 mm (1548 pixels), b = The distance from the centre of IW to the bottom of the ROI: 1 mm (960 pixels), c = The temporal offset of the centre of the ROI from the centre of the IW: 0.4 mm (384 pixels), d = Length and width of the ROI: 1.225 mm (1176 pixels).

Importantly, the IW position relative to the corneal apex varies between individuals^30^. Previous studies have reported the IW to lie approximately 1–2 mm inferior to the apex^18^ or 2.51 ± 0.23 mm inferonasal to it^27^. To accommodate this anatomical variability, the ROI was defined as a 1.50 mm^2^ (1.23 mm x 1.23 mm) square positioned with its centre 1.61 mm superior and 0.40 mm temporal to the centre of the IW (Fig. 1). This placement is expected to encompass the reported range in which the corneal apex lies relative to the IW, thereby ensuring that the apex falls within the ROI for all subjects.

Furthermore, the cornea is a curved surface, with the steepest curvature in the centre. Mapping multiple images (that are assumed to be parallel to the surface) into a planar montage will inevitably introduce geometrical distortions. Either meridian lengths are shortened or the circumferential length is stretched. The error increases with increased area to be analysed. Based on a standard curvature of central cornea, *r* = 7.7 mm, (horisontal 7.86, and vertical 7.65)^31^ and a 1.5 mm^2^ central ROI, the expected geometrical distortion error will remain below 1%, as shown in Allgeier’s dissertation^32^, and can therefore be considered negligible.

### Imaging

**Fig. 2:**
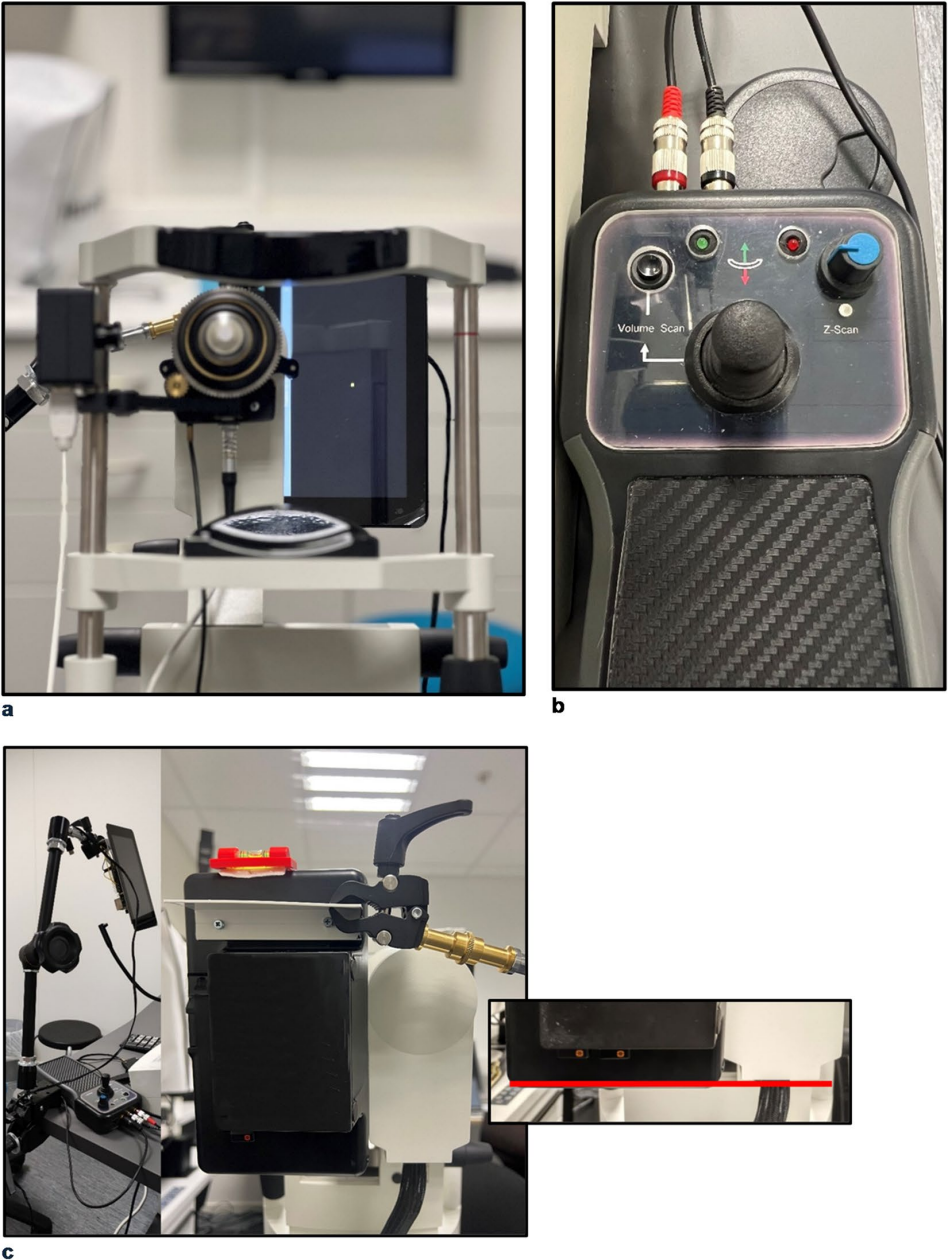
Instrument setup with the touch screen, the motorised joystick, and the levelling vial. **a** the setup from the subject’s view, looking at the white fixation dot on the screen. **b** the motorised joystick applied for depth adjustment and starting and stopping image acquisition. **c** the screen with the fixation device mounted to the table with levelling vial for horizontal alignment.

The laser scanning IVCM (HRT III with Rostock Cornea Module, Heidelberg Engineering GmbH, Heidelberg, Germany) was utilised, equipped with 63x/0.95 NA objective lens with a 400 µm x 400 µm field of view. A Raspberry Pi 4 Model B Rev 1.1 (Raspberry Pi Foundation / Raspberry Pi Holdings, manufactured by Sony UK Technology Centre, Pencoed, Wales), running with Raspberry Pi Os, with a Raspberry Pi 7” Touch Screen (Raspberry Pi Foundation, Cambridge, UK) with resolution 800 (RGB) x 480 pixels, was mounted beside the standard IVCM for presenting the fixation target (Fig. 2a, c).

The imaging protocol was developed in collaboration between the University of South-Eastern Norway (SAS, EL, TLM, VS), University of Applied Science and Arts Northwestern Switzerland (EK), Linköping University (NL), and the Karlsruhe Institute of Technology (SA). By using the IW as a reference landmark and applying a standardised fixation target, the protocol enables consistent acquisition of repeatable mosaic images of corneal nerve fibres in the corneal apex, the designated ROI.

**Fig. 3:**
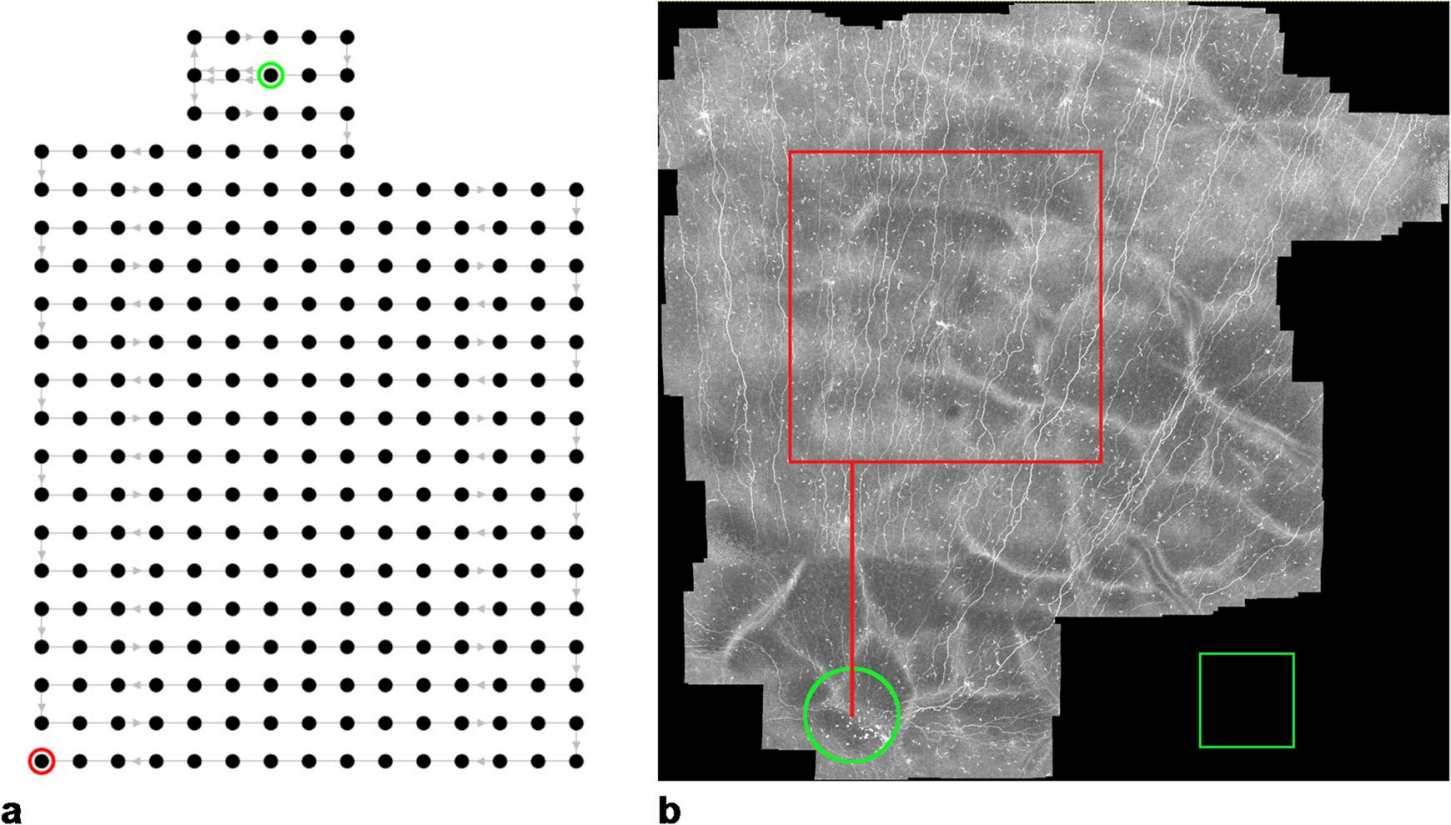
**a**. The fixation pattern; starting at the green circle and finishing at the red circle following in a predefined stepwise pattern (arrows), in total 266 fixation points. Three fixation points in line two from the top are used twice to support a visually smooth and uninterrupted fixation sequence. The fixation pattern is inverted compared to the area of final image depiction, as an inferior gaze enables imaging of the superior plexus, and vice-versa. **b**: Predefined ROI covering 1.5 mm^2^ in the left eye (red square) along with a predefined circle centred on the inferior whorl (IW) with a diameter of 400 μm (green circle). The green square represents the size of one image frame (0.4 mm x 0.4 mm).

Starting at the IW, the fixation target moved through a predefined pattern on the touch screen while images were acquired simultaneously using the instrument’s sequence scan mode. The number of fixation points and spacing between them were designed to provide sufficient image overlap, enabling construction of a continuous mosaic of the ROI within a clinically acceptable acquisition time (Fig. 3). The fixation target, a 1 mm diameter white circle, was displayed for 1 second at each of the 266 locations, with the screen positioned 240 mm from the eye. To capture the full ROI with redundancy, a total of 8–9 sequence scans were required. Sequence scanning was chosen because it is generally less operator dependent than section scanning^12,14,33^. Each sequence consisted of 100 images covering a 400 μm x 400 μm area (384 × 384 pixels), acquired at a frame rate of 3 frames/sec, resulting in theoretically 798 image frames per examined eye. Consecutive sequence scans were manually restarted upon completion of each scan.

### Image post processing

To reduce operator dependency, all images acquired during the full imaging protocol were included in the mosaic construction. A MATLAB algorithm processed single images into a full mosaic^22,25,34,35^. The algorithm establishes correspondences between overlapping parts of the acquired image data, based on the exhaustive image registration of all possible image pairings in a dataset. Using a registration quality metric, the image pair alignment results are partitioned into a subset of those that are accepted as correct and the rest that are ignored. Where the integration of all available image data into a single mosaic image is not possible, the images of a given dataset are montaged into several separate, non-overlapping or only minimally overlapping mosaic images.

**Fig. 4:**
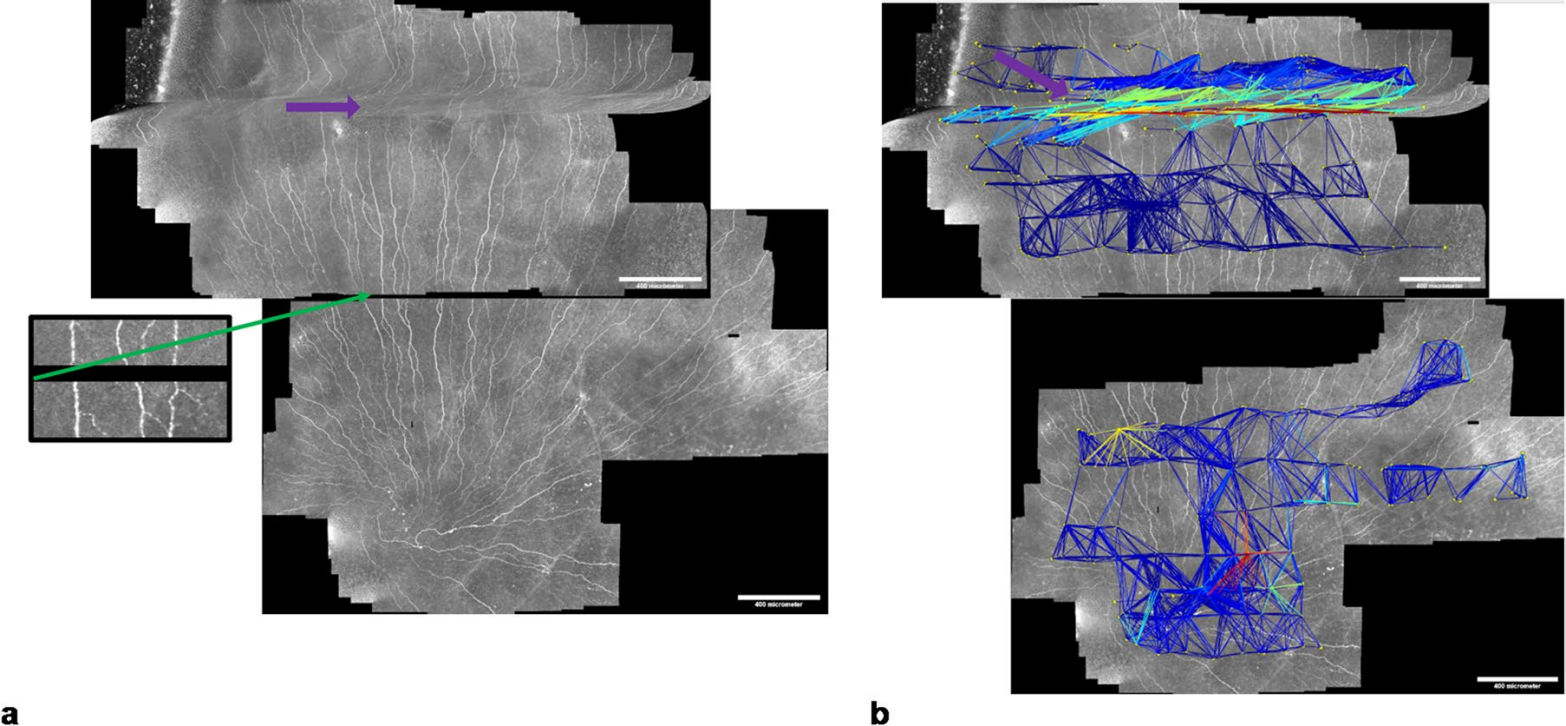
Example of strong deformation errors and an incomplete mosaic (separate mosaic segments) caused by misalignment in central region of the reconstruction. The white scale bar represents 400 μm. **(a)** The purple arrow indicates a marked deformation artefact, which corresponds to the areas highlighted by the red and yellow lines in panel (b). The green arrow marks a manually inferred alignment, illustrating where the two mosaics would likely have been correctly positioned. **(b)** Blue lines denote areas where image alignment was successful. In contrast, red and yellow lines show geometric distortions caused by incorrect motion artefact correction and misalignment. Panel **(b)** also demonstrates the gap between the two mosaic segments and shows how the blue lines fail to connect corresponding structures needed to produce a continuous mosaic.

The image mosaicking algorithm described above runs fully automated. A single, easy-to-use process parameter (continuous scalar-valued) gives the operator a means to influence the algorithm result. This parameter specifies the acceptable upper limit for the residual alignment error with respect to an individual image pair. It effectively controls the partitioning of the image pair alignment results described above by assessing the internal consistency of the accepted subset. Higher values for this parameter relax the restriction on the internal consistency requirement, which directly leads to a higher number of accepted image pair alignment results. This increases the potential to include more image data in a single large mosaic image, but at the same time increases the risk of accepting incorrect image pair alignment information, which can lead to erroneous montages, potentially containing strong geometric deformations (Fig. 4). Vice versa, decreasing the parameter value reduces the risk of such deformation errors, but goes along with an increased probability that the montaging process results in more separate smaller mosaic images. An empirically determined default parameter value of 16 has been found to work well for many datasets. The results were visually inspected after processing. If the resulting mosaic images contained readily visible, strong deformation errors (Fig. 4), the threshold parameter was gradually reduced to 8, 6, and 4, until the strong deformation errors vanished.

### Assessment of the imaging method

#### Sample

Healthy subjects between the age of 18 and 30 were recruited among students at the University of South-Eastern Norway. Informed written consent was obtained from all participants. According to the Norwegian Health Research Act (§ 2), this methodological study falls outside the scope of the Act and therefore does not require assessment by the Regional Committees for Medical and Health Research Ethics (REK/IRB). In line with the University of South-Eastern Norway’s agreement with – the Norwegian Agency for Shared Services in Education and Research (SIKT), ethical and data protection aspects were reviewed and approved by SIKT (Ref. no. 249925). The study adhered to the tenets of the Declaration of Helsinki.

### Patient setup and image acquisition

Both eyes were anesthetised with Oxibuprocaine 0.5%, using two drops in the eye being examined and one drop in the eye used for fixation. Additionally, a 2 mg Carbomer viscous gel was applied as a coupling agent between the TomoCap and the examined eye. To minimise discomfort and ensure stable fixation, a drop of lubricant (Thealoz duo, Thea Pharmaceuticals, Clermont-Ferrand, France) was instilled in the fixating eye.

Vertical alignment of the IW was done by manually moving the fixation target prior to the image acquisition and using the adjustment knobs for refinement. The image acquisition was started when the centre of IW was visible in the real-time IVCM software display window. During the examination, the focus plane was adjusted at moderate speed by a motorised joystick attachment (Fig. 2b). To ensure good imaging quality and sufficient area horizontally, the contact between the TomoCap and the cornea was continually adjusted. Additional details regarding the image acquisition protocol are provided in the Supplementary Materials, section 1, p 2-7.

Each eye was imaged three times. A five‑minute interval between acquisitions allowed participants to sit back and rest between sessions. To facilitate the assessment of intra- and inter-operator agreement, the first operator (either SAS or EL) performed the imaging twice, while the second operator (either EL or SAS) performed it once. The order of the operators was alternated between subjects. Both operators were optometrists, trained and proficient in the use of IVCM.

### Image quality assessment

**Fig. 5:**
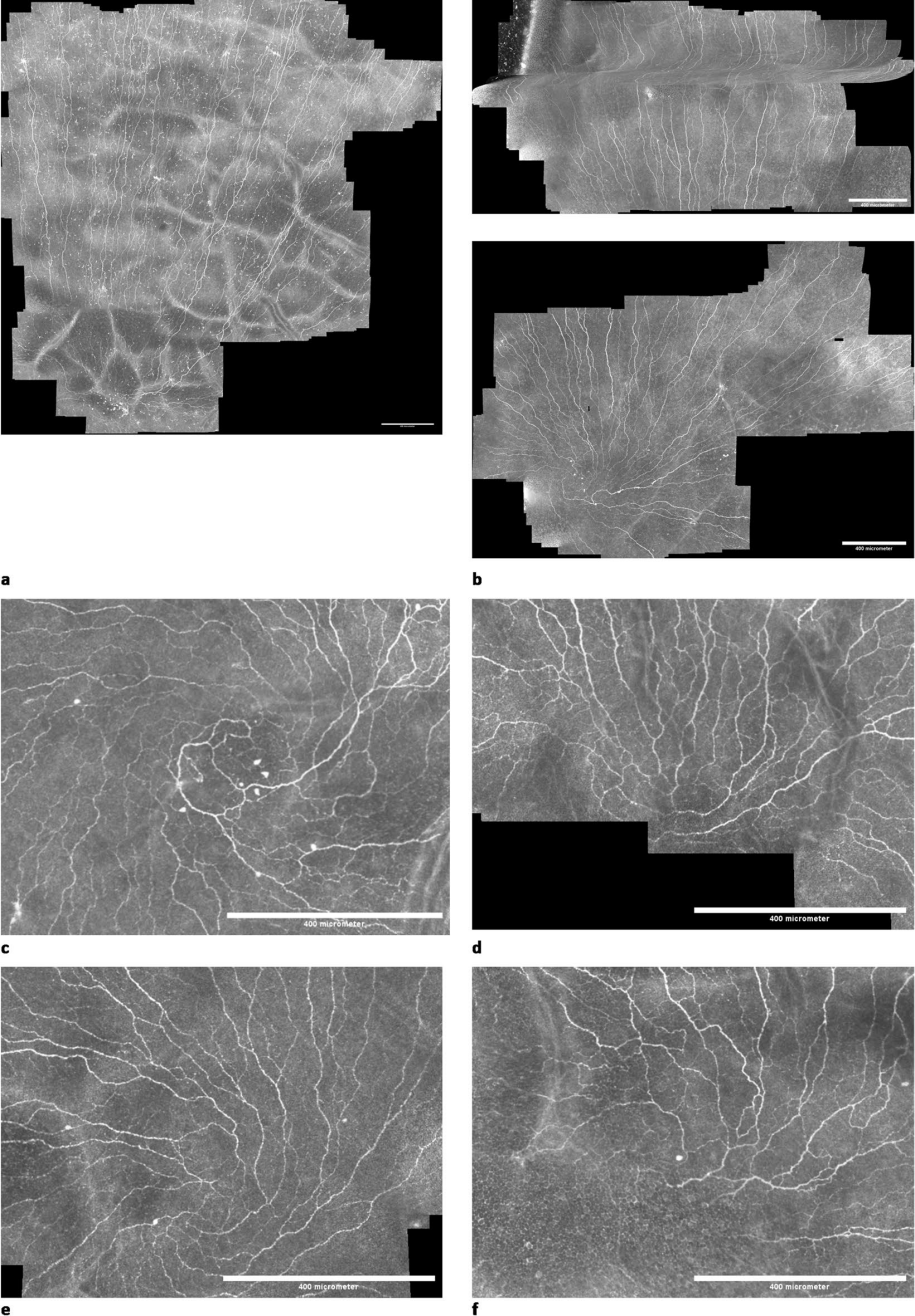
Illustrations of inclusion and exclusion within the three domains of the image quality grading. Domain 1; mosaic continuity and identification of the inferior whorl (**a-b**), domain 2; completeness of inferior whorl region imaging (**c-d**), and domain 3; image quality and identification of the inferior whorl centre (**e-f**). For more details see supplementary material, section 2, p 8-13) The panels to the left (**a**, **c** and **e**) show examples classified as grade 1 (include), whereas the panels to the right (**b**, **d** and **f**) show examples classified as grade 0 (exclude).

The IW (Fig. 3b, green circle) served as the reference region for evaluating imaging quality because of its distinct and easily identifiable nerve‑fibre pattern. The image quality of all complete mosaics was assessed independently by four graders (VS, EL, ES, and BMA), who were masked to information about the participant, eye laterality, imaging order, and operator, thereby ensuring unbiased assessment. *Image quality* was assessed using a predefined, three-domain grading framework (Supplementary Materials section 2, p 8-13) The domains were: (1) mosaic continuity and identification of the IW, (2) completeness of imaging of the IW region, and (3) image quality within the IW region. Each domain was scored dichotomously as Include (1) or Exclude (0) based on standardised visual criteria referenced to example images. A total quality score (0–3) was calculated by summing scores across domains. Images with score < 3 were excluded from further analysis (Fig. 5). Borderline cases were graded conservatively, with anatomical identifiability of the IW prioritised over overall image quality. In cases of disagreement, a consensus review group (SAS, TLM, VS and EL) reviewed the images together and reached an agreement regarding inclusion in the analysis.

For each eye, the mosaic with the highest quality depiction of the IW, termed the reference mosaic, was used to define the centre of the IW. If two or more images were considered to be equal quality, the mosaic in which the IW was most horizontally centred, with identifiable structures inferior to the centre, was selected as the reference mosaic. In cases where the location of the whorl was equally good across mosaics, the mosaic with the highest contrast was chosen.

### Identification of the reference point and extraction of region of interest

The protocol for defining the centre of IW, used as the *reference point*, was based on previously published qualitative descriptions of the whorl^27^. For the reference mosaic, three operators (JL, SAS, and EK) independently determined the centre of the IW. The corresponding x and y coordinates were recorded in ImageJ^36^ following a detailed protocol (Supplementary Materials section 3, p 14-16). To enable assessment of both inter- and intra-operator agreement, one operator (SAS) determined the centre of the IW twice, while the two other operators (JL and EK) each performed it once.

Considering the individual variability in anatomy and the subjectivity involved in precisely defining a centre point, certain discrepancies were deemed acceptable, and the Euclidean distance (hypotenuse) between these points was calculated. This distance is represented as a vector, combining the differences in the x and y pixel coordinates between the two points (x_1_, y_1_) and (x_2_, y_2_) using the Eq. (1):

1 $$\:d=\:\sqrt{({\mathrm{x}2\:-\:\mathrm{x}1)}^{2}+{(y2-y\:1)}^{2}}$$

This distance serves as a measure of the positional discrepancy between the two operators.

For further analysis, the first reference point placed by the operator who completed the center determination twice (SAS) was used, ensuring consistency across the repeated assessments. Subsequently, the centre of IW was transferred to the other mosaics for the same eye by aligning them with the reference mosaic in GIMP (GNU Image Manipulation Program, version 2.10.12, The GIMP Development Team, 2019, https://www.gimp.org)^37^.

A MATLAB script (MATLAB version 24.1.0.2689473 R2024a. Update 6; The MathWorks, Inc, Natick, MA, USA) used the specific coordinates for the centre of IW for each mosaic to calculate the percentage of ROI imaged, hereafter called the *ROI area coverage*, and the percentage of the 400 μm diameter circle around IW, hereafter called the *centre IW area coverage*.

### Statistical analysis

Statistical analyses were performed using Excel for Windows, version 2411 (Microsoft Corporation, Redmond, WA, USA) and IBM SPSS Statistics, Version 29.0.1.0 (IBM Corp., Armonk, N.Y., USA). Shapiro-Wilk test (*n* < 50) was used to evaluate the normality distribution of the data. Descriptive analysis parameters were expressed as the mean and standard deviation for normally distributed continuous variables, median and interquartile range for non-normally distributed data, and frequency for discrete variables. The Wilcoxon signed‑rank test was used to assess differences in IW and ROI area coverage between the two image‑acquisition operators.

Fleiss’ kappa was used to assess inter and intra-rater agreements for nominal categorical variables. For variables with ordered categories, weighted kappa with quadratic weights was used, as this statistic accounts for the magnitude of disagreement between raters. A kappa value of 1 indicates perfect agreement, whereas a value of 0 reflects agreement no better than chance. According to the criteria of Landis and Koch, κ values of 0.21–0.40 indicate fair agreement, 0.41–0.60 indicate moderate agreement, 0.61–0.80 indicate substantial agreement, and values above 0.80 indicate almost perfect agreement^38^. Confidence intervals (95%) were calculated for both kappa statistics. A two-way mixed effect, single measures, absolute agreement intraclass correlation coefficient (ICC) was calculated to assess the inter- and intra-operator agreement for the x and y coordinate of the centre of IW^39^. Results were considered statistically significant when p < α, with α set to 0.05.

## Results

### Datasets for inclusion

A total of 72 datasets were acquired from 24 eyes, with three sequences obtained per eye. The mean (± SD) image acquisition time was 397 (± 52) seconds per dataset. For 65 datasets, a valid mosaic image was achieved, of which 56 datasets, at the default threshold level of 16. For nine of the 65 datasets, the threshold value was adjusted to 8 (*n* = 5), 6 (*n* = 2), or 4 (*n* = 2), respectively.

**Fig. 6:**
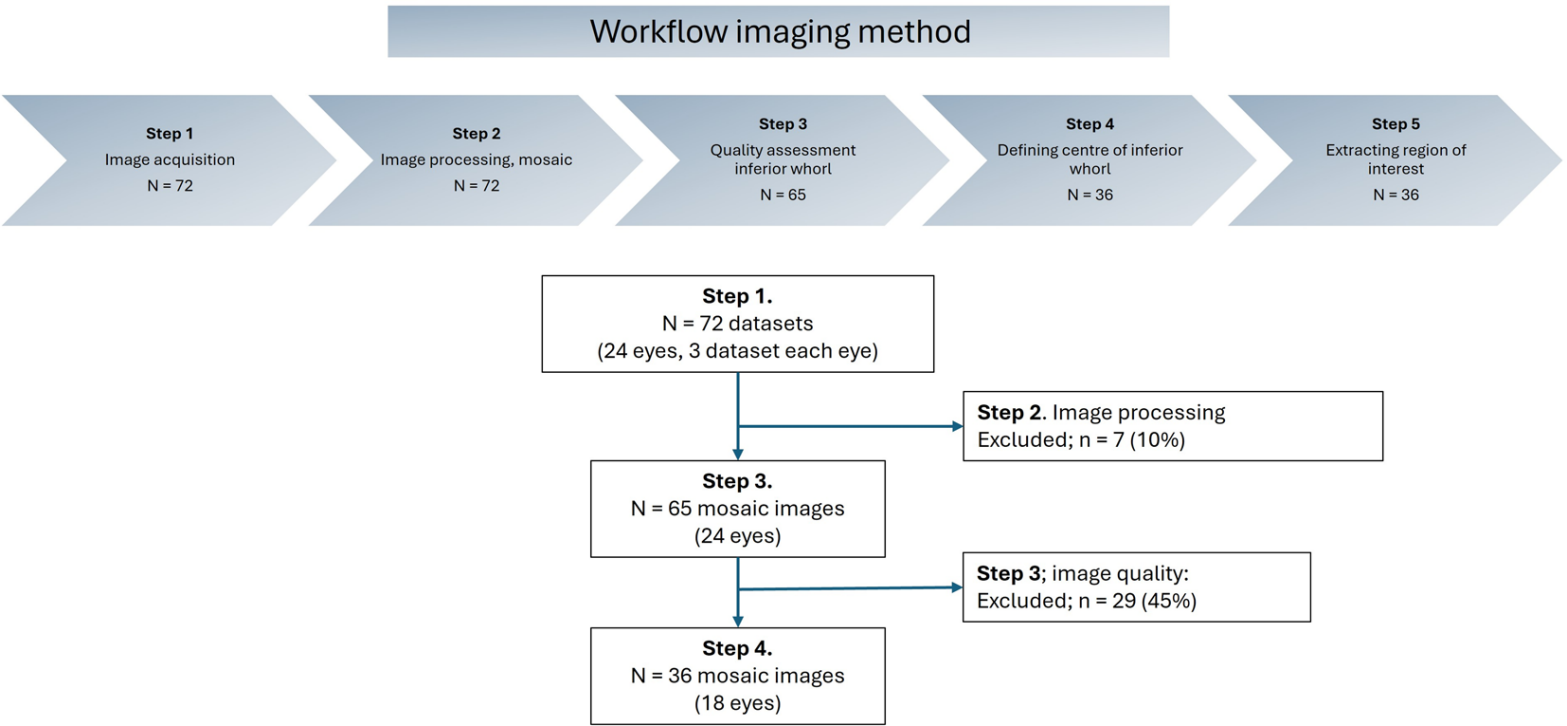
The arrow panel illustrates the workflow from image acquisition through image processing, quality assessment, placing the centre of the inferior whorl, and extracting the ROI. The flowchart below illustrates how many datasets, mosaics and eyes that were included and excluded throughout the workflow. In the image processing (step 2), two datasets were excluded due to processing errors and five because of a non-continuous mosaic between inferior whorl and the ROI. In step 3, 29 mosaics were excluded because of unmet image quality criteria.

The workflow for excluding mosaics that did not meet the intended standard of quality is presented in Fig. 6. In total, seven datasets (10%) were excluded because of failure to achieve a complete mosaic. Five of the datasets (7%) were excluded due to the absence of continuous mosaic image including both the central corneal area and the IW. The other two datasets (3%) were excluded because it was not possible to generate a mosaic free of strong deformation errors, even at threshold level 4.

### Image quality

Of the 65 complete mosaics, the two operators imaged 30 (46%) and 35 (54%), respectively. In total, 36 (55%) mosaics had acceptable image quality for identifying the IW and were used to define the IW centre. Adequate image quality was obtained for all three mosaics of 5 eyes; 8 eyes had two gradable mosaics, and 5 eyes had 1 gradable mosaic. Among the 29 excluded mosaics, six eyes (from five different participants) had no gradable mosaics, reducing the number of eyes available for further analysis to 18.

For the IW region quality assessment, interrater agreement (Fleiss’ kappa) for (1) mosaic continuity and identification of the IW, (2) completeness of imaging of the IW region, and (3) image quality within the IW region was fair to moderate, with κ = 0.361 (95% CI [0.164, 0.497]), 0.410 (95% CI [0.246, 0.556]), and 0.447 (95% CI [0.292, 0.600]), respectively. The overall image inclusion demonstrated moderate agreement, κ = 0.540 (95% CI [0.398, 0.664]), *p* < .001.

### Identification of the reference point

Excellent inter- and intra-rater agreement was demonstrated for the identification of the IW centre. The median (IQR) inter-rater vector length difference for OP1 vs. OP2, OP1 vs. OP3, and OP 2 vs. OP3 were 34 (80), 57 (40), and 39 (42) pixels, respectively. The intra-rater vector length difference was 18 (28) pixels. The inter-rater agreement for the x and y coordinates assessed using ICC (two-way mixed effects model, absolute agreement single measures, 95% CI) was 0.996 (95% CI [0.991, 0.999]) and 0.997 (95% CI [0.994, 0.999]), respectively. The intra-operator agreement was 0.999 (95% CI [0.997, 1.000]) and 1.000 ((95% CI [0.999, 1.000]) for x and y coordinates.

### Region of interest coverage

In 34 of 36 (95%) of the mosaics the 400 μm diameter circle around the IW was completely imaged, (Table 1). There was no statistically significant difference in IW coverage between the two operators (Wilcoxon signed rank test, z = -1.000, *p* = .317). In 22 of 36 (61%) of the mosaics, the ROI was completely imaged. The difference in ROI coverage between the two imaging operators was not significant (Wilcoxon signed rank test, z = 0.135, *p* = .893). Among the mosaics with a non-complete imaging, the missing area of the ROI occured on the temporal side of the cornea (6 of 36, 16%).

**Table 1:** Overview of the 18 eyes and their ROI coverage and centre of IW coverage by operators.

Eye ID	ROI (%) M1	ROI (%) M2	ROI (%) M3	IW (%) M1	IW (%) M2	IW (%) M3	OP # schedule
1	100	-	87^a, b^	100	-	100	1, -, 2
2	97^b, c^	100	-	100	100	-	2, 2, -
3	-	100	75^d^	-	100	100	-, 2, 1
4	100	100	100	100	100	100	1, 2, 1^f^
5	-	-	100	-	-	100	-, -, 2
6	100	90^a^	-	100	100	-	2, 2, -
7	100	100	100	100	100	100	1, 1, 2
8	81^a^	-	-	100	-	-	2, -, -
9	94^c^	100	100	100	100	100	2, 2, 1
10	-	100	100	-	100	98^e^	-, 1, 2
11	100	-	100	100	-	100	1, -, 2
12	100	100	100	100	100	100	2, 2, 1
13	96^a^	93^a^	94^a^	100	100	100	2, 2, 1
14	-	-	100	-		100	-, -, 2
15	100	79^c^	-	100	100	-	1, 1, -
16	-	87^d^	-	*-*	98^a^	-	-, 2, -
17	-	100	-	-	100	-	-, 2, -
18	-	16^d^	68^d^	-	100	100	-, 1, 2

^a^missing parts of ROI temporal, ^b^missing parts inside ROI, ^c^missing parts nasal, ^d^missing parts superior, ^e^missing parts inferior ^f^4th examination replacing an examination due to error, Bold numbers = OP1, Italic numbers = OP2. Abbreviations: ROI; region of interest, MI1-3; measurement number 1–3, IW; inferior whorl, OP; operator.

## Discussion

This pilot validation study evaluated a proposed method for standardised imaging of corneal subbasal nerves using IVCM, with the aim of improved consistency of imaging of the SBNP at the corneal apex. Across most datasets, the central ROI was fully and repeatedly captured with minimal inter- or intra-operator difference. To our knowledge, this is the first study to investigate imaging of a predefined central ROI using a standardised imaging method and to assess the repeatability of image capture.

### Technical innovation and reproducibility

The proposed imaging method offers several advantages over previously published approaches^9–14^. First, the ROI was deliberately defined to be larger to account for the well-known inhomogeneous distribution of the corneal nerve fibres. Second, the unique structural landmark of IW^17,27,28^ was used as a reference point, facilitating consistent relocation of the same ROI in subsequent examinations and thereby minimizing spatial sampling error and improving the stability of repeated measurements. Third, all images (approximately 798 images, including 266 fixation points) were included in the mosaicking process, in which the central area (ROI) corresponded to a 15 × 16 grid (240 positions). A larger sampling area is expected to enhance the robustness of quantitative metrics and reduce selection bias^23,24^.

Prior studies support the importance of both sampling area and image selection. Using the IW as reference point and acquiring one section-image per position, Takhar et al.^13^ reported good to excellent inter-operator repeatability for CNFL (mm/mm^2^) when employing a 5 × 6 grid (30 positions). However, repeatability declined when the grid was reduced to six images (3 × 2)^13^. In the present study, the sampling density was substantially higher: the 240 central fixation points used represent almost an eightfold increase compared with the 30-position grid in Takhar et al., which likely contributed to the stability of repeated ROI imaging by reducing spatial under-sampling and improving coverage of the central subbasal nerve plexus.

Other published work has shown that image selection also influences reliability^12,29^. Kalteniece et al. demonstrated excellent repeatability across operators when a strict image-selection protocol was applied^29^, whereas Schaldemose et al. found substantial variability when 3–6 images were sampled at random^12^. Human image selection is therefore a recognised source of bias that increases variability and reduces reproducibility. The approach used in the present study addresses these limitations by eliminating image selection entirely and by standardising both the sampling area (ROI) and spatial reference (IW).

The present study demonstrated excellent inter- and intra-rater agreement for identifying the spatial reference (IW). In most included mosaics, both the IW region (defined as a 400‑µm diameter circle around the IW centre) and the ROI were fully captured. Overall, the method provides a reproducible imaging strategy that minimises operator dependency and enhances technical precision in corneal nerve assessment. The repeatability for CNFL derived from this approach will be investigated in future studies.

### Clinical implications

The present work evaluated the feasibility of consistently imaging the same region of the SBNP by different operators across multiple imaging sequences. This capability is particularly important for longitudinal studies and for clinical follow-up examinations, where imaging the same area over time is essential for monitoring disease progression or treatment response. In contrast, previous studies have primarily focused on the repeatability and reliability of quantitative measurements derived from fixed image datasets^10,13,15,29,33,40^. To account for the variability and inhomogeneity of nerve fibres in the central cornea^19,23^, the total ROI area in the present study was set to 1.5 mm^2^, providing an anatomically referenced, repeatable and larger ROI than those reported in other imaging protocols^12,14,19,23^. Multiple studies have examined how the number of images acquired affects the reliability of quantitative outcomes^24,41^ and demonstrate that relying on capturing a single image from the same corneal region over time yields poor repeatability^10^. A comprehensive literature review indicates that most studies involving IVCM in people with diabetes and control groups typically analyse 3–6 images^17^, a number that may not adequately represent the true CNFL of the SBNP when compared with larger mosaics^22^. This limitation is particularly relevant in conditions with reduced nerve fibre density, including older age and diabetes, where analysing CNFL in a small area can lead to greater variability and reduced reliability^42^. In such populations, a larger, anatomically consistent ROI is especially important to ensure more stable and representative measurements.

The larger, anatomically consistent, and repeatable ROI used in the current study addresses key limitations of previous approaches and provides a stronger foundation for more accurate and reproducible CNFL quantification, thereby demonstrating feasibility and supporting the potential for more robust clinical application of IVCM. Furthermore, the method relies on low‑cost, easily accessible software and hardware; the python code for the fixation target is openly available^43^, along with the fixation pattern^44^ and the software used to create the mosaic^45^.

Although the method shows promise for enabling a more standardised and reproducible approach to corneal nerve imaging, its applicability is currently limited by the small sample size and the pilot‑stage nature of the study. While the findings offer early methodological insight, validation in larger and more diverse cohorts is necessary before any clinical implementation can be justified.

### Future perspectives

We acknowledge that there are methodological limitations and areas for improvement. One limitation is that image acquisition was completed by only two operators. Although the use of a standardised image acquisition protocol likely reduces operator dependency, including a larger number of operators would enable a more robust assessment of the method’s reliability. Future studies should therefore involve multiple independent operators to confirm the generalisability of our findings. In addition, the inter- and intra-rater agreement for both IW and ROI should be interpreted with caution, given the selected sample and limited sample size.

It is also important to note that, although the IW is a unique anatomical landmark, it is dynamic, and the mechanisms governing its orientation are not fully understood^46^. Evidence suggests that the anatomical orientation of the whorl may change with age, indicating a need for longitudinal studies to understand how the centre of IW and associated structures evolve over time^47^. Improving our understanding of these changes is essential for ensuring consistent ROI definition and maintaining accuracy in long-term clinical assessments.

In the current study, 11% of dataset were excluded after mosaic processing. In the MATLAB mosaicking procedure used to determine the relative alignment between any two given images, one image is decomposed into twelve horizontal slices (sub-images), each of which is registered to the second image. A minimum of three consistent relative alignment results among these twelve sub-images are required for the alignment to be considered as valid; if this criterion is not met, all sub-image alignment results between the respective image pair are discarded^48^. To reduce mosaic exclusion due to image quality and incomplete data, and to ensure successful mosaicking between the IW and central cornea, several adjustments can be implemented. These include increasing the acquisition frame rate to improve image overlap, enhancing image quality, reducing processing errors, and improving vertical alignment. The optimal frame rate will depend on the specific software and hardware configuration and should be selected to balance adequate image overlap against total image acquisition time and the number of images requiring processing.

A total of 38% of the mosaics were excluded due to inadequate image quality of the SBNP in the IW region, primarily because poor contrast and overlying structures obscured the centre of the whorl. This high exclusion rate highlights the sensitivity of IVCM image acquisition to both operator technique and depth control. General image quality can be improved by ensuring optimal coupling between the eye and microscope, including generous application of viscous gel and stable applanation pressure with correct alignment with the cornea, factors that typically improve with operator experience. Depth control is particularly critical, as IVCM detects light originating only from the focal plane. Imaging at an incorrect depth, even slightly above or below the SBNP, introduces blur and artefacts from epithelial or stromal layers^49^. As a result, small variations in axial depth readily produce overlying structures that obscure fine details in the center of the IW. In the present study, the images were captured as two-dimensional sequence scans, even though the corneal nerve fibres follow a three-dimensional course. All captured images were combined into mosaics, including those containing information from adjacent layers, which reduced the visibility of the SBNP. As a result, our reproducibility assessment was limited to the x–y plane, and variability in the z-dimension was not evaluated. Ensuring consistent focal-plane positioning during acquisition would likely reduce these artefacts and improve visualization of the inferior whorl. Acquiring volumetric stacks, three-dimensional scans, at each position could further mitigate depth-related limitations, although this would be more time-consuming and technically demanding in routine clinical protocols^22^. Future work should explore how depth consistency and 3D acquisition strategies influence image quality, nerve visibility, and quantitative reproducibility, particularly in anatomically complex regions such as the inferior whorl.

The fixation pattern, which allows the fixing eye to align the imaged eye with the IVCM tracking area, can be adjusted to follow a vertical rather than a horizontal raster pattern. A vertical raster pattern can improve control of the vertical overlap of successive images and therefore enhance overall mosaic quality. This benefit arises from the design of the mosaicking algorithm: vertically adjacent images need to overlap by at least three sub-images, or 25% of the image height whereas horizontally adjacent images may overlap by less than 25% of the image width, provided that at least three sub-images overlap partially.

Hardware configuration also influences fixation performance. Specifically, attaching the fixation target directly to the instrument may promote more consistent and reliable fixation. Micro-saccades and involuntary eye movements remain a challenge during IVCM image acquisition, particularly when imaging in a predefined fixation positions using a fixation grid^18^. To our knowledge, fixation stability during IVCM has not been quantitatively characterized in the existing literature. However, fixation-guided approaches have shown the potential to improve the consistency of image acquisition. For example, systems such as the EyeGuidance system use a computer-controlled moving fixation target to guide gaze and enable acquisition of large area IVCM mosaics within a short time^9,11^. Despite its advantages, this system is not available to most research groups. In the present study, we employed sequence scans and facilitated more controlled fixation by presenting a dynamic fixation target to the non-examined eye, thereby supporting stable and reproducible acquisition. Although quantitative fixation stability metrics have not been reported for IVCM specifically, such metrics are well established in other ophthalmic imaging modalities and could be adapted for use in future IVCM methodology development.

Despite several limitations, including limited knowledge about the dynamic nature of the IW, only two image acquisition operators, the limited number of eyes, and remaining opportunities to enhance mosaic construction, image quality and fixation control, the method demonstrates clear strengths. The imaging approach offers advantages in contexts where repeatability is essential, such as longitudinal studies or multi-centre clinical trials in which datasets are acquired by different operators. It shows strong clinical feasibility and improves over previous strategies for image acquisition and selection. Reduced operator dependence further supports the potential of IVCM to serve as a reliable clinical tool, although continued refinement is needed to ensure image quality and complete ROI coverage.

By providing source code and open accessible resources, this work establishes a foundation for ongoing methodological development and clinical application. The approach has potential for integration in larger studies examining additional aspects of the corneal nerve morphology and function, such as corneal sensitivity, ocular pain, peripheral neuropathy, dry eyes, and other diseases where the nerve fibres are affected.

## Conclusion

The novel, standardised imaging method presented in this study indicates that the inferior whorl may serve as an effective anatomical reference point for imaging of the corneal subbasal nerve plexus. By anchoring the region of interest to the inferior whorl, the approach enables more repeatable and anatomical consistent imaging, supporting improved comparability across examinations. While the method shows promise, further refinement and technical development are required to enhance its reliability and robustness, particularly for broader application of in vivo confocal microscopy examination of corneal subbasal nerves.

## Supplementary Information

Below is the link to the electronic supplementary material.

Supplementary Material 1

Supplementary Material 2

Supplementary Material 3

Supplementary Material 4

Supplementary Material 5

Supplementary Material 6

Supplementary Material 7

Supplementary Material 8

Supplementary Material 9

Supplementary Material 10

Supplementary Material 11

Supplementary Material 12

Supplementary Material 13

Supplementary Material 14

Supplementary Material 15

Supplementary Material 16

Supplementary Material 17

Supplementary Material 18

Supplementary Material 19

## Data Availability

The data supporting the findings of this study are not publicly available at this time as they will be used in future publications. Access to the data may be granted upon reasonable request, to corresponding author, and subject to conditions that ensure the integrity of ongoing research.

## Declarations

### Patient and public involvement

Patients and/or the public were not involved in the design, conduct, reporting, or dissemination plans of this research. However, participants were recruited and took part in the study as research subjects during data collection.
