# Supplementary figures and images for: A novel method for standardised imaging of corneal subbasal nerves by in vivo confocal microscopy – a pilot validation study
Source: Sci Rep. 2026 Jun 9;16:22682. doi: 10.1038/s41598-026-54268-8 (PMC13385856; doi:10.1038/s41598-026-54268-8)

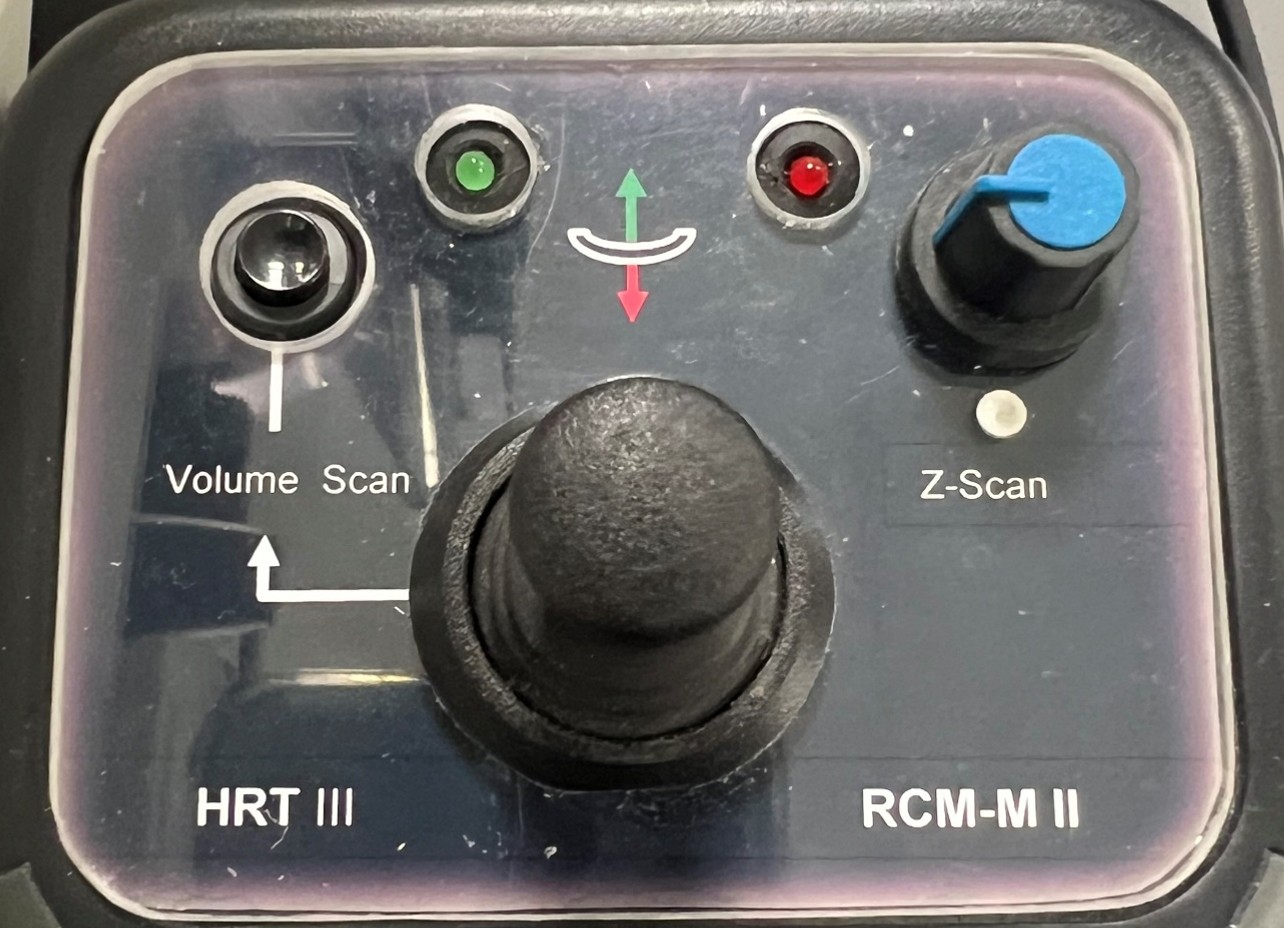

Supplement: Supplementary file 1 — Supplementary Material 1 [file 41598_2026_54268_MOESM1_ESM.jpg]

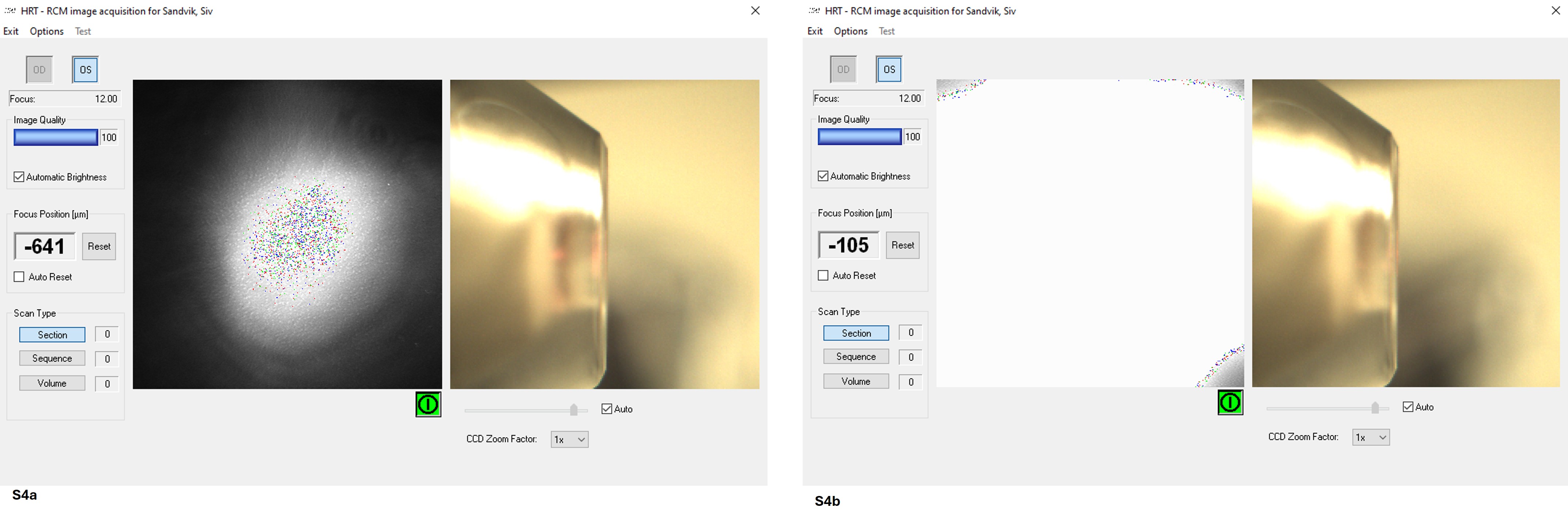

Supplement: Supplementary file 2 — Supplementary Material 2 [file 41598_2026_54268_MOESM2_ESM.jpg]

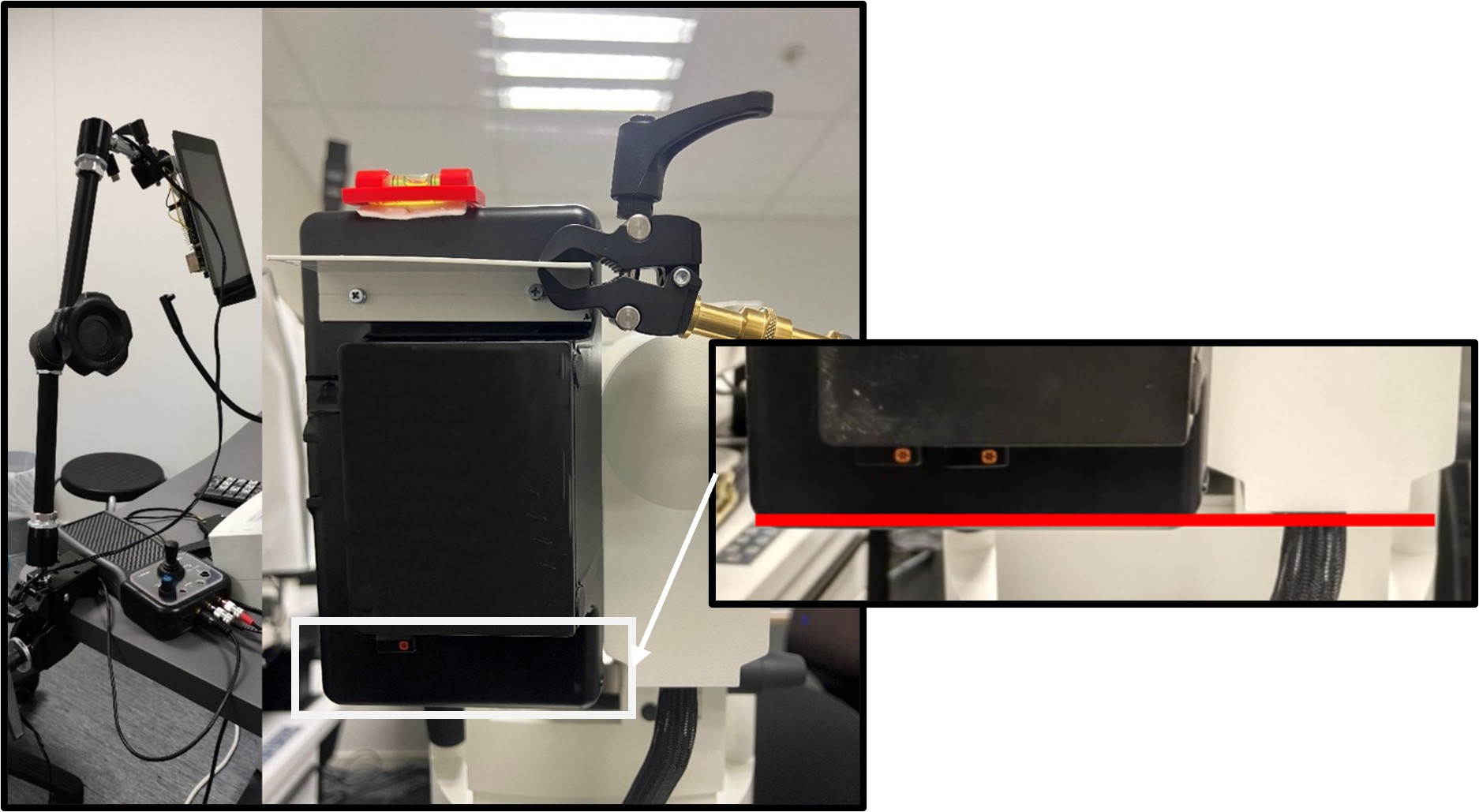

Supplement: Supplementary file 3 — Supplementary Material 3 [file 41598_2026_54268_MOESM3_ESM.jpg]

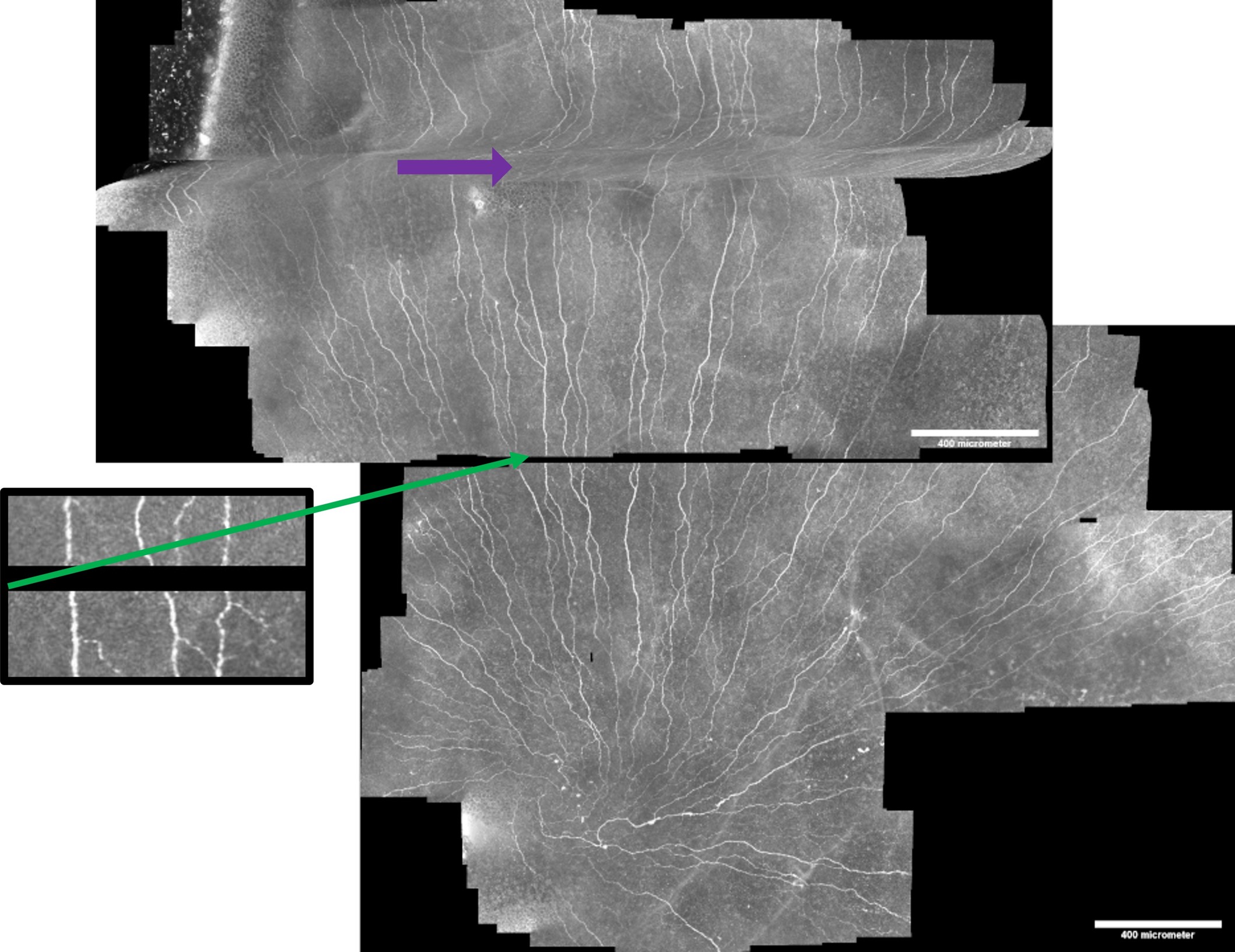

Supplement: Supplementary file 4 — Supplementary Material 4 [file 41598_2026_54268_MOESM4_ESM.jpg]

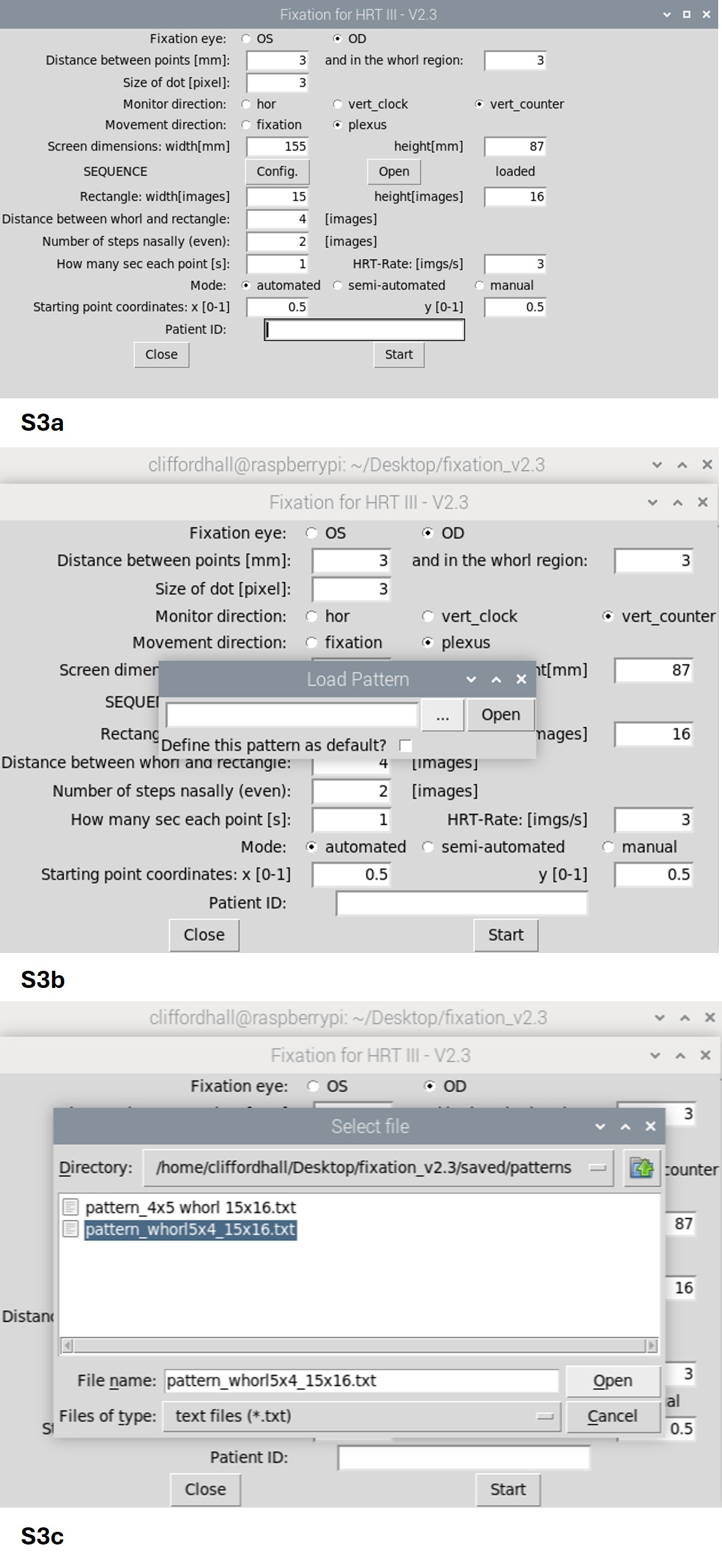

Supplement: Supplementary file 5 — Supplementary Material 5 [file 41598_2026_54268_MOESM5_ESM.jpg]

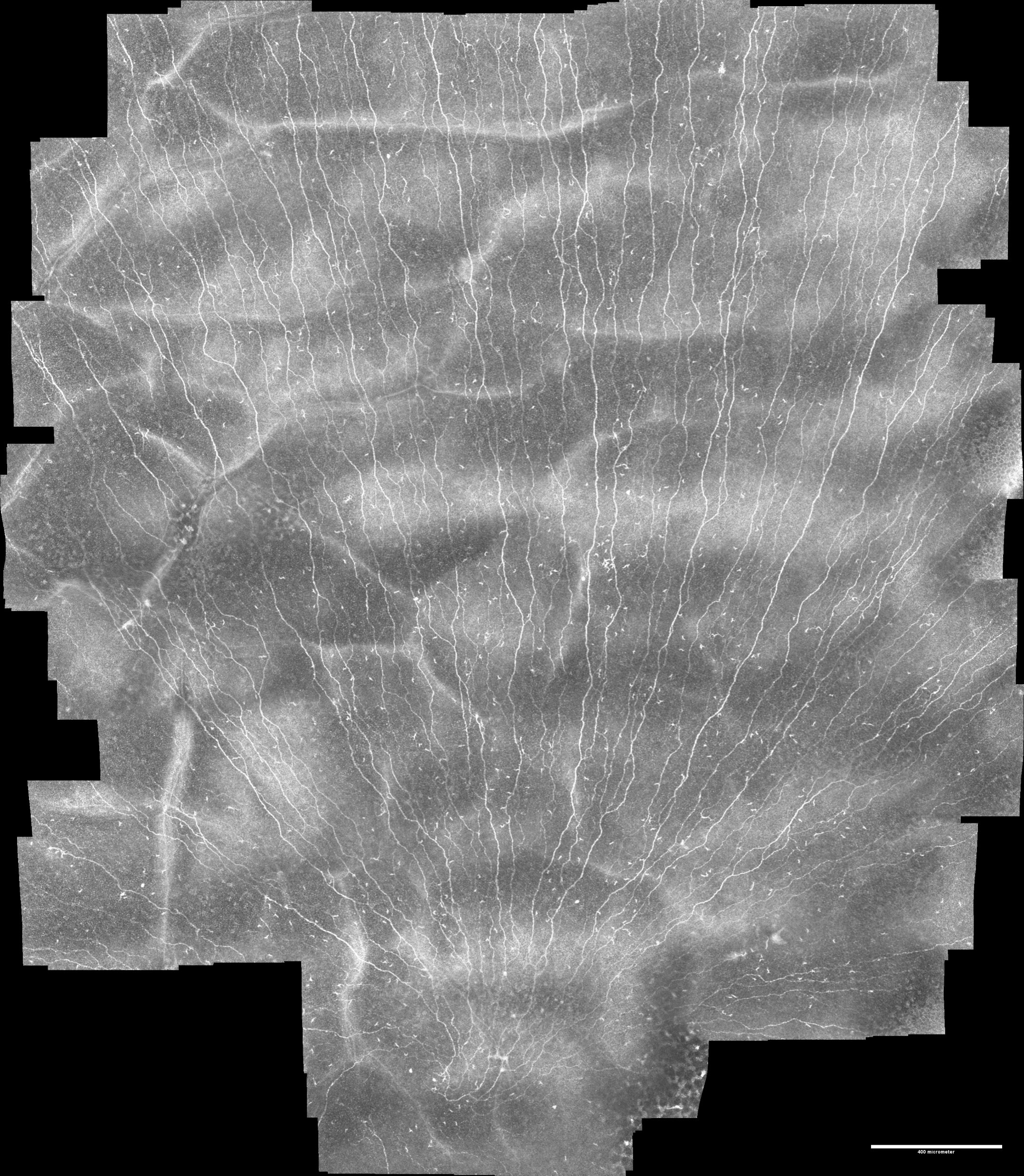

Supplement: Supplementary file 6 — Supplementary Material 6 [file 41598_2026_54268_MOESM6_ESM.jpg]

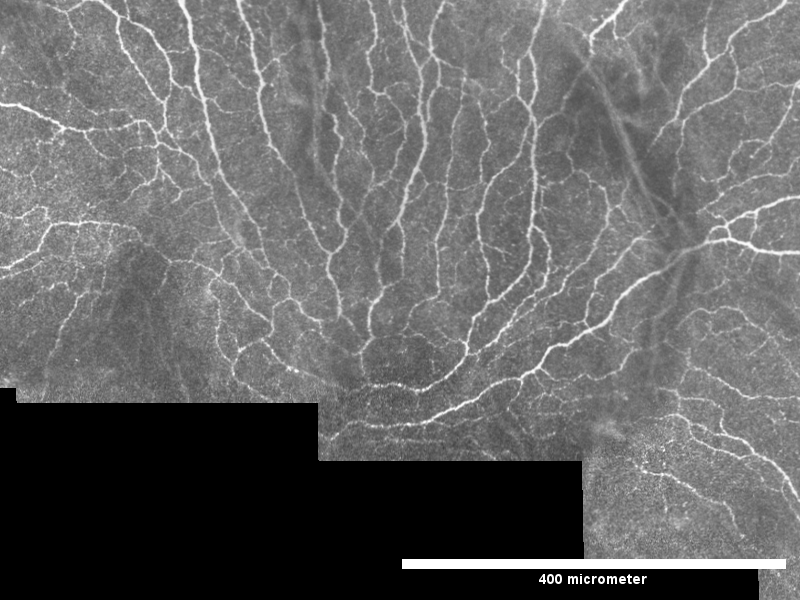

Supplement: Supplementary file 7 — Supplementary Material 7 [file 41598_2026_54268_MOESM7_ESM.tif]

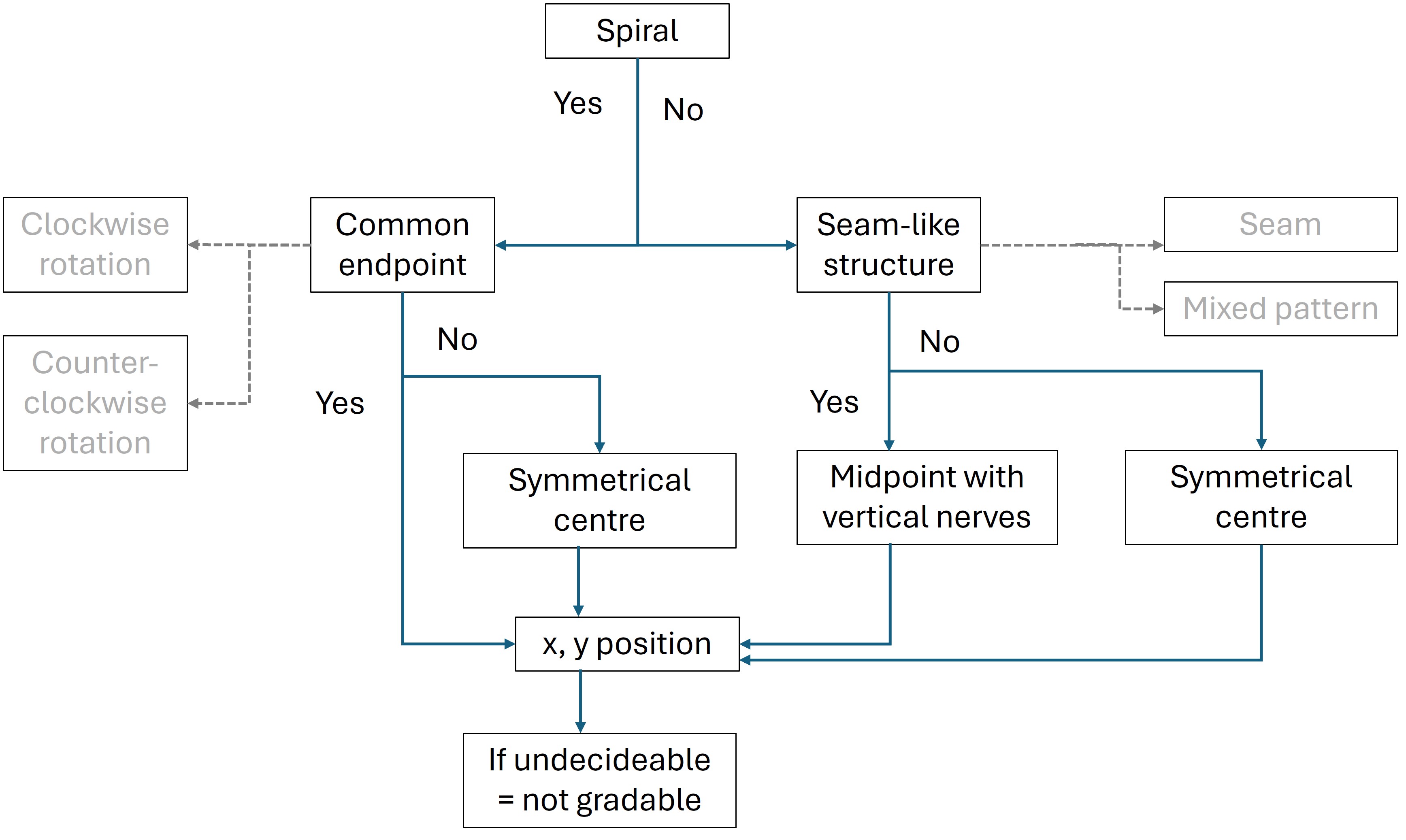

Supplement: Supplementary file 8 — Supplementary Material 8 [file 41598_2026_54268_MOESM8_ESM.jpg]

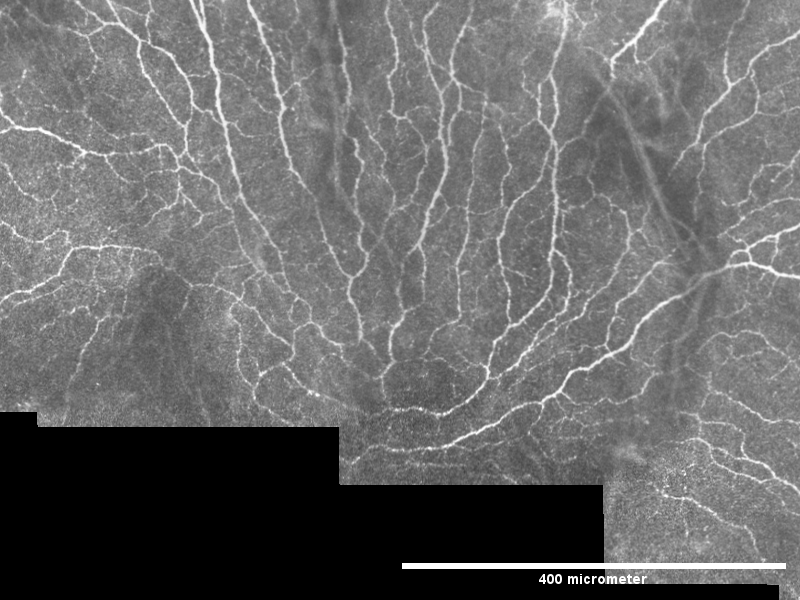

Supplement: Supplementary file 9 — Supplementary Material 9 [file 41598_2026_54268_MOESM9_ESM.tif]

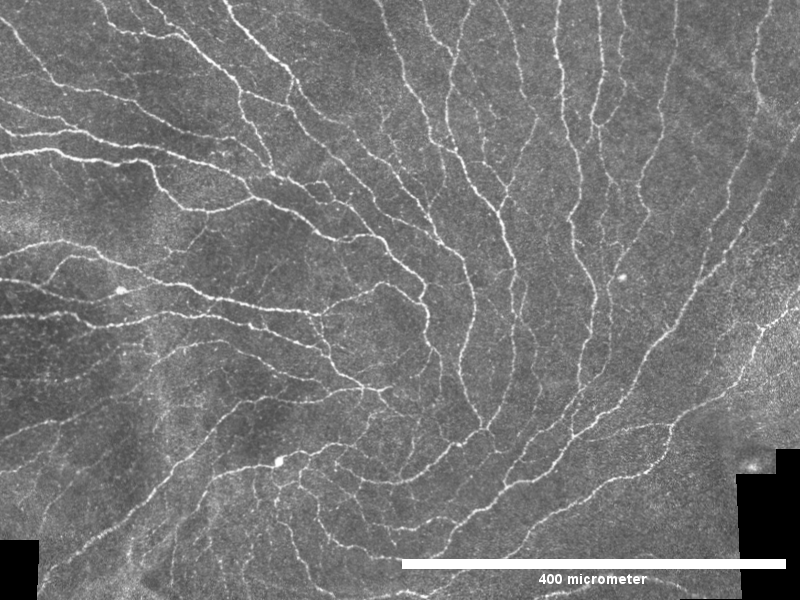

Supplement: Supplementary file 10 — Supplementary Material 10 [file 41598_2026_54268_MOESM10_ESM.tif]

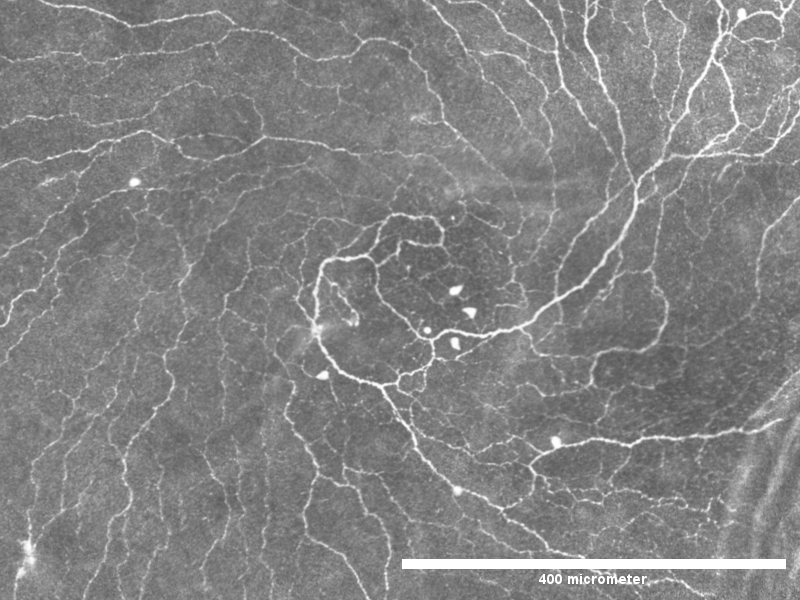

Supplement: Supplementary file 11 — Supplementary Material 11 [file 41598_2026_54268_MOESM11_ESM.tif]

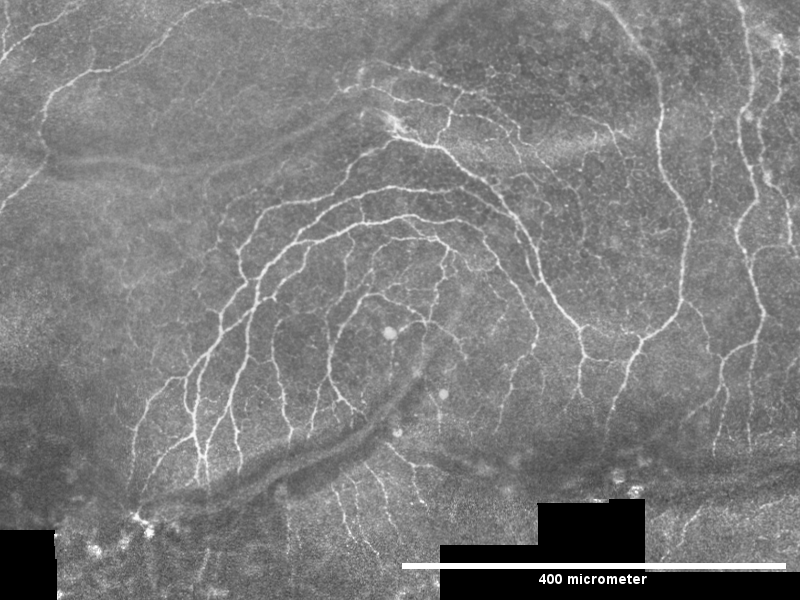

Supplement: Supplementary file 12 — Supplementary Material 12 [file 41598_2026_54268_MOESM12_ESM.tif]

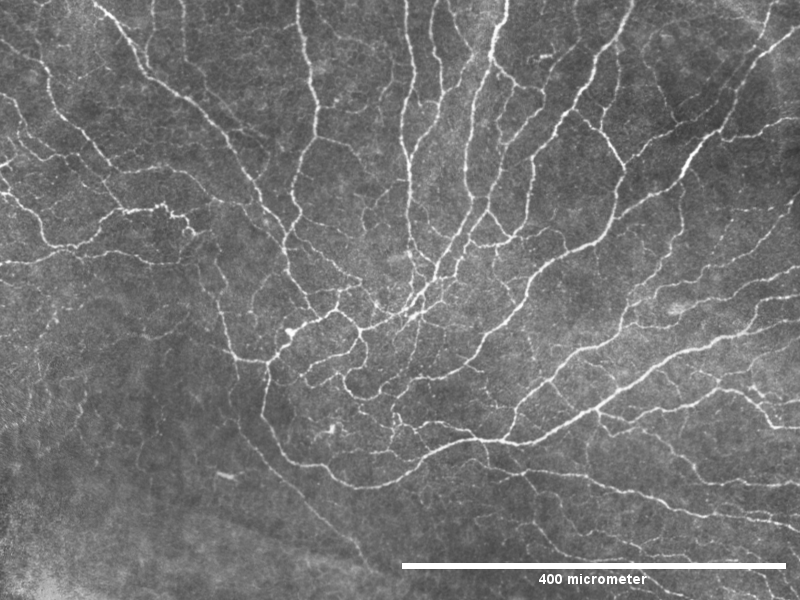

Supplement: Supplementary file 13 — Supplementary Material 13 [file 41598_2026_54268_MOESM13_ESM.tif]

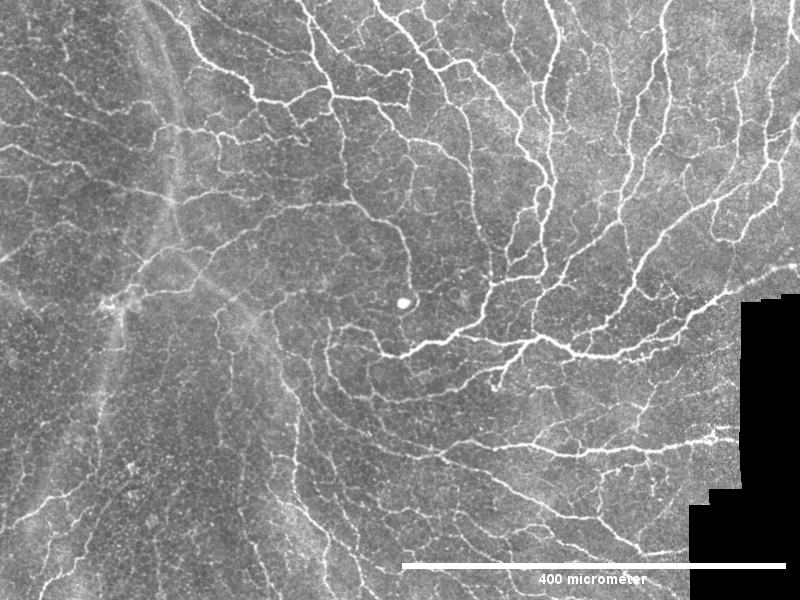

Supplement: Supplementary file 14 — Supplementary Material 14 [file 41598_2026_54268_MOESM14_ESM.tif]

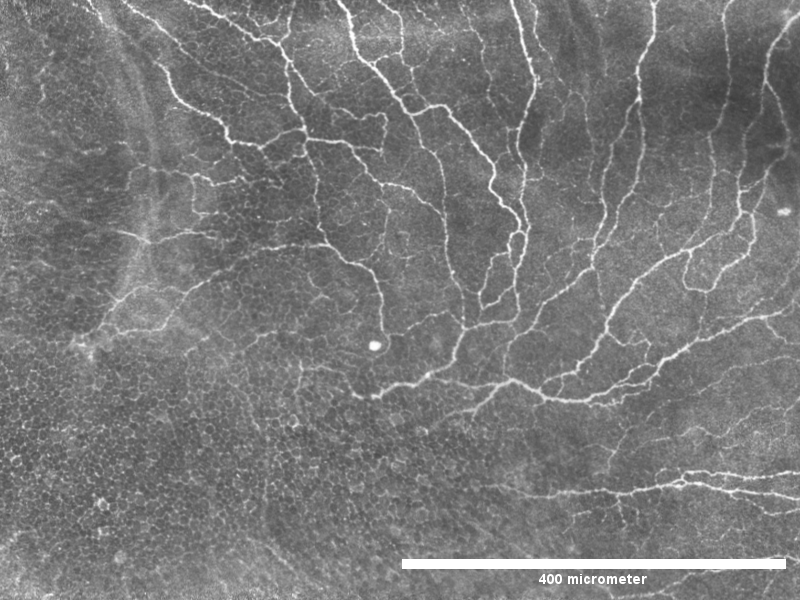

Supplement: Supplementary file 15 — Supplementary Material 15 [file 41598_2026_54268_MOESM15_ESM.tif]

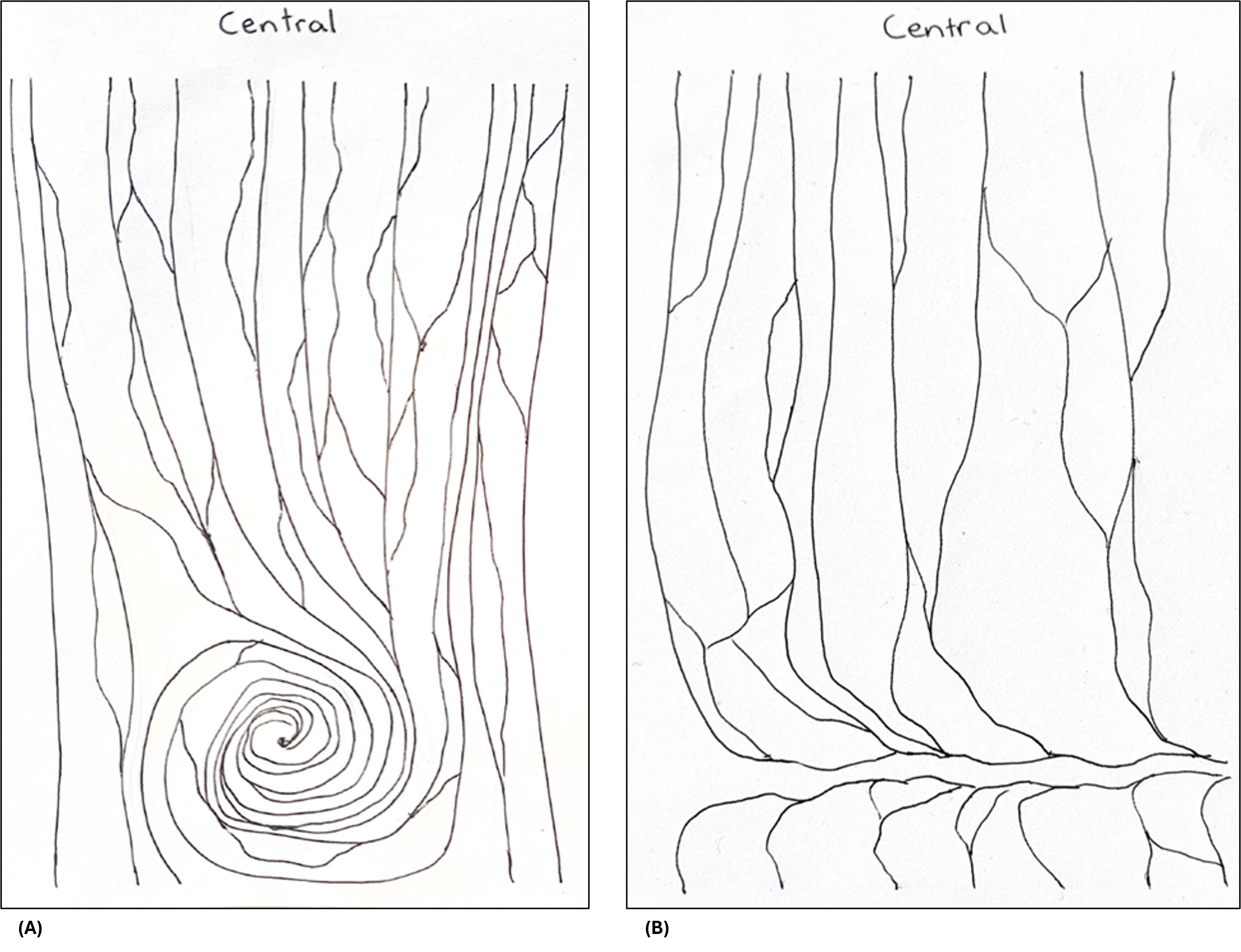

Supplement: Supplementary file 16 — Supplementary Material 16 [file 41598_2026_54268_MOESM16_ESM.png]
