# Supplementary material for: A novel method for standardised imaging of corneal subbasal nerves by in vivo confocal microscopy – a pilot validation study
Source: Sci Rep. 2026 Jun 9;16:22682. doi: 10.1038/s41598-026-54268-8 (PMC13385856; doi:10.1038/s41598-026-54268-8)
Supplement: Supplementary file 17 — Supplementary Material 17 [file 41598_2026_54268_MOESM17_ESM.docx]

# Supplementary Material

**Article Title**:

A novel method for standardised imaging of corneal subbasal nerves by in vivo confocal microscopy – a pilot validation study

**Authors:**

Siv A. Sandvik _University of South-Eastern Norway_

Eilin Lundanes _University of South-Eastern Norway_

Stephan Allgeier _Karlsruhe Institute of Technology_

Emanuele Käser _University of Applied Sciences and Arts Northwestern Switzerland FHNW_

Neil Lagali _Linköping University_

Jorunn Lid _University of South-Eastern Norway_

Tove Lise Morisbakk _University of South-Eastern Norway_

Vibeke Sundling _University of South-Eastern Norway_

## Supplementary Materials content:

| 1. Procedure for In Vivo Confocal Microscopy Imaging | Pages 2-7 |
| --- | --- |
| 1. Identification of Inferior Whorl and Image Quality Grading | Pages 8-13 |
| 1. Defining Centre of Inferior Whorl | Pages 14-16 |

## 1. Procedure for In Vivo Confocal Microscopy Imaging

**Instrument:** Heidelberg Retina Tomograph III with Rostock Cornea Module (HRT-RCM)

### 1. Preparations

#### 1.1 Turn on devices

1. Switch on the computer and the Heidelberg HRT-RCM according to the **HEYEX Manual**^[1,2]^.
2. Launch the **HEYEX software** and log in.
3. Position the **Z-scan** **speed** on the motorized joystick as shown in **Figure S1**.

|  | 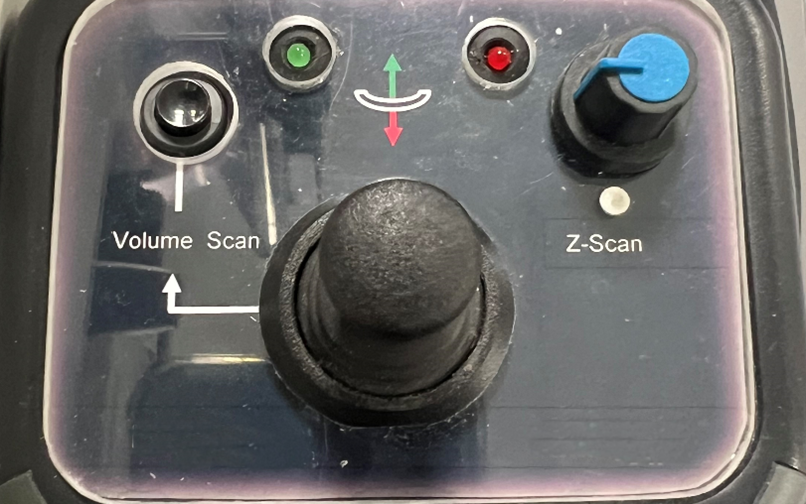  **Figure S1**. Position of the z-scan speed on the motorized joystick as indicated by the blue knob. |
| --- | --- |

#### 1.2 Prepare the fixation target (LCD screen)

1. Move the HRT at the **left endpoint**, away from the chin rest (for imaging the left eye)
2. Attach the screen to the **arm mounted on the table** (Figure S2) and position the screen in front of the fixating eye. Ensure that the screen is:
   - **Parallel** to the back of the instrument head, with the bottom edge aligned to the bottom of the instrument head (Figure S2).
   - **Level**, using the leveling vial attached to the screen (Figure S2).
3. Ask the patient to confirm that the screen is **fully visible** to the fixating eye.


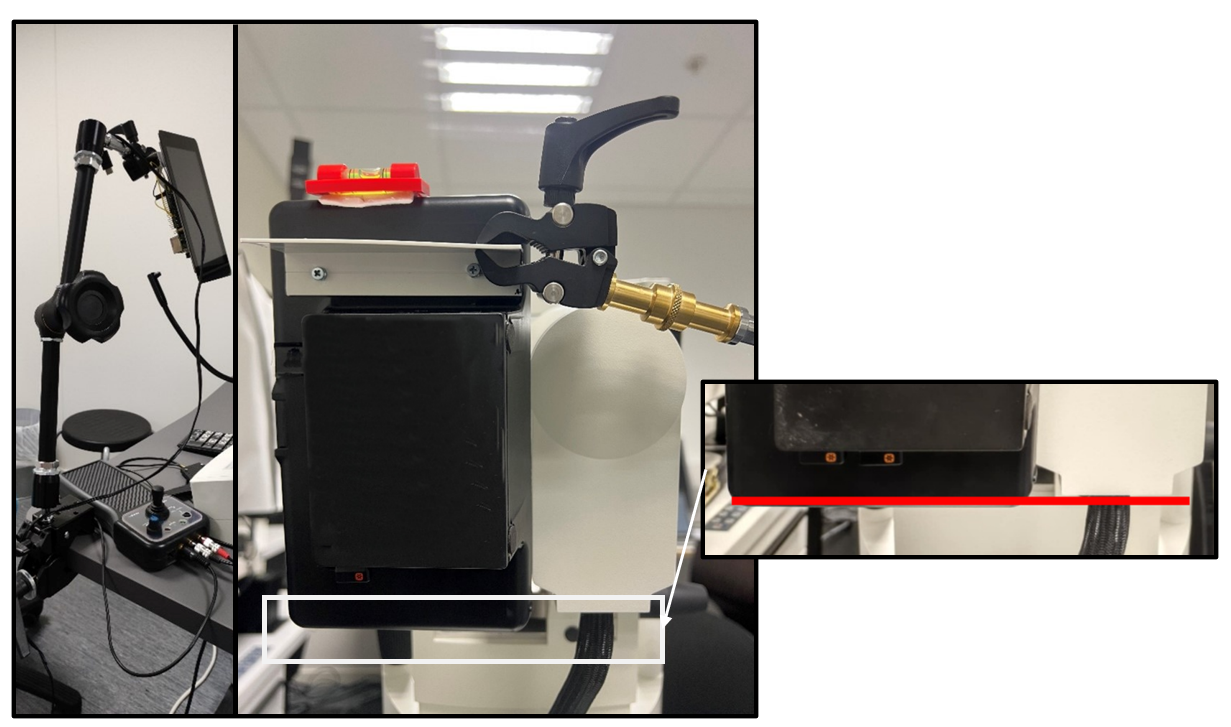


**Figure S2.** The LCD screen mounted to the table and positioned parallel to the back of the instrument head with the bottom of the screen aligned to the bottom of the instrument head. The red leveling vial on top of the screen should be used to ensure the level of the screen.

#### 1.3 Configure the Raspberry Pi

1. **Power on** the Raspberry Pi and open a **terminal window**.
2. **Start the Fixation software** (Fixation_v2.3)^[3]^:
   - Navigate to the directory Fixation_v2.3**/** and start the software using the command: cd Desktop/Fixation_v2.3/
   - Start the software with the command: python3 main.py
3. Verify that the **main menu window** appears (Figure S3a).
4. **Configure the following settings** (Figure S3a)
   - Fixation eye: **OS/OD**
   - Distance between points: **3**
   - Size of dot: **3**
   - Screen direction: **vert_counter**
   - Movement direction: **plexus**
   - Screen dimensions: **155 mm x 87 mm**
   - Sequence: Click **Open.**
     - **Load Pattern:** Click **Open** and select a pre-configured pattern (e.g., pattern_whorl5x4whorl_15x16, Figure S3b and S3c)^[4]^.
5. **Set parameters** (Figure S3a):
   - How many seconds each point: **1 second**
   - HRT rate: **3 images/sec**
   - Mode: **Automated**
   - Starting point coordinates: **x = 0.5, y = 0.5**

| 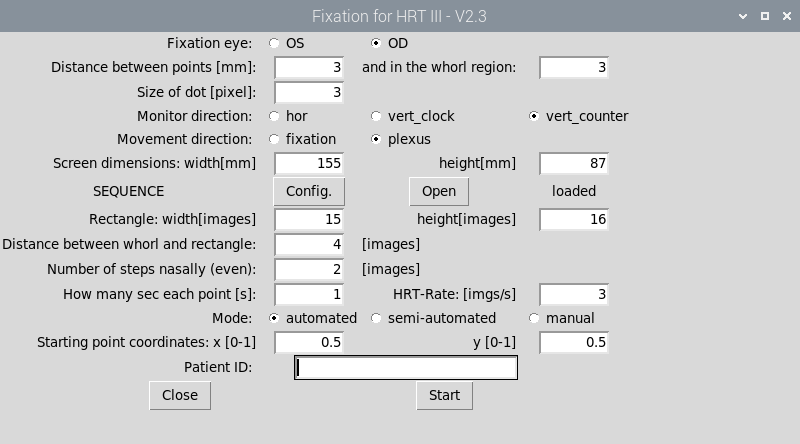  S3a  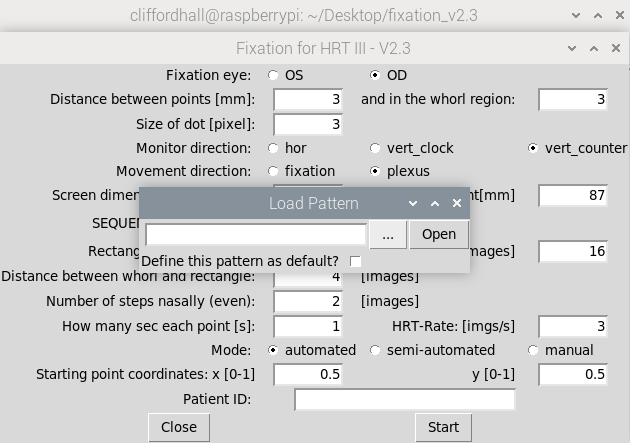  S3b  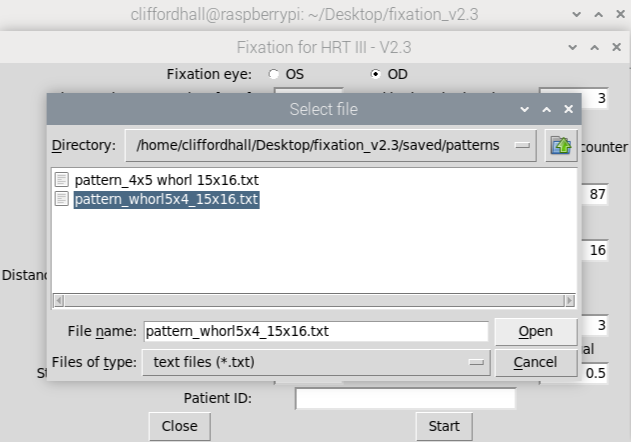  S3c |
| --- |

**Figure S3** Main menu and settings in the Fixation software: S**3a** Raspberry Pi main menu window. S**3b-c** Steps for opening the pre-configured fixation target pattern.

### 2. Patient Registration and Instrument Setup

1. **Register the patient** in the HEYEX software according to the HEYEX User Manual^[1]^.
2. **Configure the cornea module**:
   - Field of View (FOV): **400** **µm**
   - Position the Charge Couple Device (CCD) camera **90° temporal** to the eye being examined.
3. **Attach a TomoCap** to the RCM microscopy lens as described in the user manual.
4. **Adjust the RCM focal point** to the **front surface of the TomoCap** (Figure S4).

| 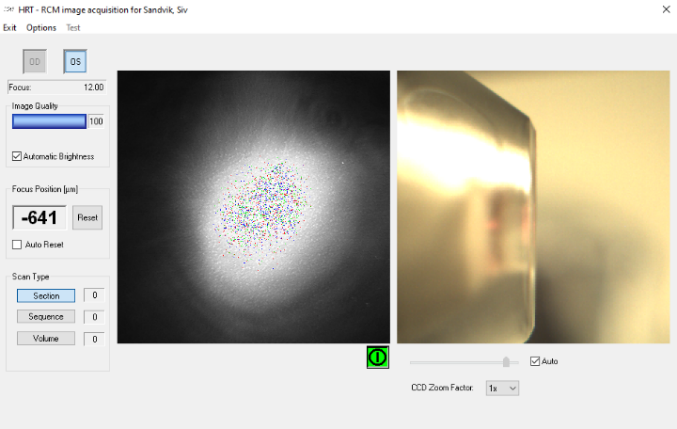 S4a | 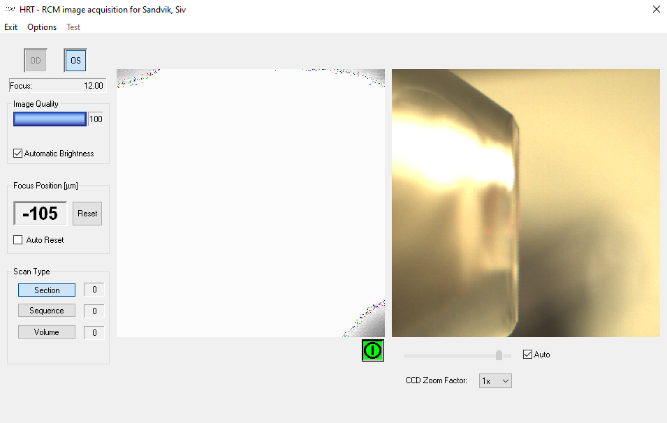  S4b |
| --- | --- |

**Figure S4**. **Focus adjustments before examination**: **S4a** Move the focus as far as possible away from the TomoCap, then advance until the first white light appears. **S4b** Continue forward until the second white light fills most of the screen**,** then reset the focus depth to zero.

### 3. Patient preparations

1. **Adjust the table height** for a comfortable, stable position:
   - Chin on chinrests, forehead firmly against forehead rest.
   - Feet flat on the floor, hands rest on thighs.
2. **Align visual axis with optical axis**:
   - Adjust headrest so eye height matches the **red alignment line**.
   - Verify CCD camera is **perpendicular** to the optical axis.
3. **Position TomoCap** about **1 cm from the cornea**:
   - Center the **red reflex** in the pupil to prepare perpendicular contact.
4. **Apply anesthetic and gel**:
   - Two drops of 0.5% **Oxibuprocaine**, 30 seconds apart.
   - Apply a pea-sized amount of viscous gel (e.g., **Visc-Ophtal** or **Viscotears**).
   - For the non-examined eye: One drop of Oxibuprocaine + artificial tears to prevent dryness.
5. **Confirm screen visibility** and ensure it is unobstructed by the instrument head.

### 4. Image Acquisition

1. **Instruct the patient** to fixate on the target displayed on the screen.
2. **Create touch between the TomoCap and central cornea**:
   - Center the **red reflex** in the pupil using the adjustments knobs (see Heidelberg User Manual).
   - Move the instrument toward the cornea while monitoring the red reflex on the computer screen.
   - Corneal touch is confirmed when **corneal structures become visible** on the screen.
   - Move the fixation target superior to **locate the inferior whorl**.
3. **Start the imaging:**
   - Start a **sequence** **scan**.
   - Press **Enter** on the Raspberry Pi to initiate the fixation pattern.
   - **Restart sequence scans** as needed until the fixation pattern is complete.
4. **To maintain optimal clarity of the sub-basal nerve plexus throughout the image acquisition:**
   - Use the **motorized joystick** for fine focus adjustments.
   - Use the **camera adjustment knob** for fine pressure adjustments.

## 2. Identification of Inferior Whorl and Image Quality Grading

### 1. Evaluation and scoring

Three domains are evaluated and scored in the following order:

1) Mosaic continuity and identification of the inferior whorl,

2) Completeness of imaging of the inferior whorl region, and

3) Image quality and identification of the inferior whorl centre

Each criterion is scored dichotomously (Include = 1 point; Exclude = 0 points). Images with a total score < 3 are excluded for further analysis.

| **Domain** | **Include (1 point)** | **Exclude (0 points)** |
| --- | --- | --- |
| **1. Mosaic continuity and identification of the inferior whorl** | The mosaic image provides continuous anatomical coverage from the central cornea to the inferior whorl region, demonstrated by subbasal nerve fibres extending inferiorly and converging toward a common endpoint, either as a whorl (spiral) or a seamlike anatomical nerve pattern. (Fig. S5A, B & S6a) | The mosaic image does not provide continuous anatomical coverage from the central cornea to the inferior whorl region, thereby precluding identification of the inferior whorl. (Fig. S6d) |
| **2. Completeness of inferior whorl region imaging** | The inferior whorl region can be identified, with no or only minor loss of image information (e.g. small gaps or poor contrast) that does not impede identification. (Fig. S6b) | The inferior whorl region cannot be identified due to moderate or severe loss of image information (e.g., extensive gaps or poor contrast) that impedes identification. (Fig. S6e) |
| **3. Image quality and identification of the inferior whorl centre** | The centre (or axis) of the inferior whorl region can be identified despite the presence of additional structures, such as pressure lines, minor motion artefacts, epithelial or stromal cells, provided the sub-basal nerve pattern can be followed towards the whorl centre. (Fig. S6c.) | The centre (or axis) of the inferior whorl region cannot be identified because additional structures, such as pressure lines, motion artefacts, epithelial cells, or stromal cells, obscure the sub‑basal nerve pattern, preventing reliable identification. (Fig. S6f) |

### 2. Scoring of borderline cases

Borderline cases arise when image characteristics partially meet inclusion criteria but introduce uncertainty regarding inferior whorl identification. To ensure consistency, objectivity, and reproducibility, the following decision rules should be applied. When scoring borderline images, identifiability takes precedence over image aesthetics. An image should be included if the required anatomical feature can be identified with reasonable confidence based on nerve pattern continuity and orientation, even in the presence of minor artefacts. Images should be excluded if uncertainty remains after systematic assessment.

#### 2.1. Decision Rules for Borderline Cases

1. **Mosaic continuity and identification of the inferior whorl (Domain 1)**

**Include** if a continuous inferior trajectory of sub‑basal nerve fibres can be visually traced from the central cornea to a convergence point as a whorl/spiral or seam‑like pattern consistent with the inferior whorl, even if minor gaps are present.

**Exclude** if the nerve trajectory is interrupted between the central cornea and the inferior whorl region, or if the inferior whorl region cannot be confidently confirmed.

**Rule**: Include if nerve fibre continuity from central cornea to inferior whorl is visually demonstrable; otherwise exclude.

1. **Completeness of inferior whorl imaging (Domain 2)**

**Include** if the inferior whorl region can be localized despite small gaps, limited field of view, or reduced contrast.

**Exclude** if missing regions or diffuse poor contrast prevent localization of the inferior whorl region.

**Rule**: Include if the inferior whorl region can be localized despite minor image information loss; otherwise exclude.

1. **Image quality and identification of whorl centre (Domain 3)**

**Include** if the sub‑basal nerves can be followed towards a visible centre or axis of convergence, despite the presence of pressure lines, minor motion artefacts, epithelial or stomal cells.

**Exclude** if additional structures or artefacts obscure nerves such that the centre or axis of the inferior whorl cannot be identified with confidence.

**Rule**: Include if nerve fibres can be followed to a visible inferior whorl centre or axis; otherwise exclude.

#### 2.2. Tie‑Breaking and Consensus

In cases of uncertainty, the more conservative score (Exclude) should be assigned. If grading is performed by multiple raters, borderline cases should be resolved by consensus review or adjudication by a senior rater. Borderline exclusions should be logged to support transparency and interrater reliability analysis.

| 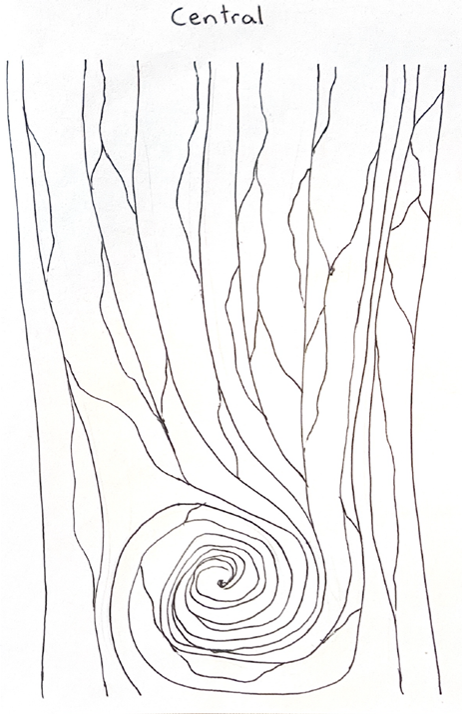 | 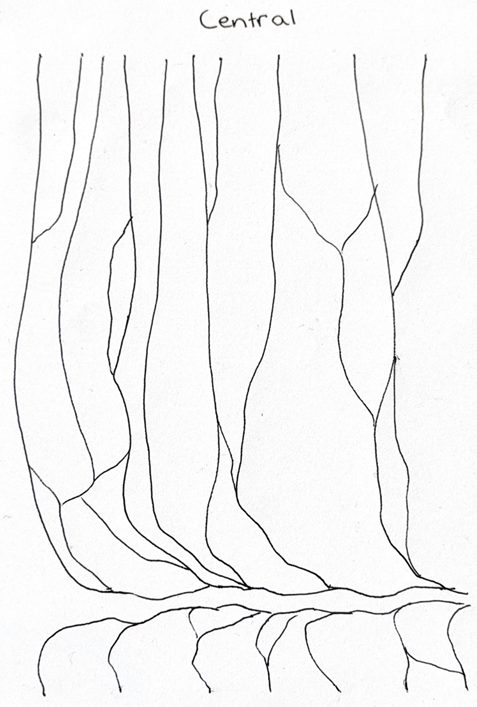 |
| --- | --- |
| (A) | (B) |

**Figure S5. Anatomical variants of the inferior whorl region used for grading.** Representative schematic and exemplar images illustrating valid anatomical configurations of the inferior whorl region of the sub‑basal nerve plexus. Sub-basal nerve fibres can be visually traced from the central cornea to a region of convergence identifying the inferior whorl, appearing as either (A) whorl-like convergence, or spiral, towards a single centre or (B) seam-like convergence along a linear axis. Both configurations were considered acceptable for checklist-based identification; seam refers to nerve architecture, not a mosaic boundary. Illustration: Emilie Sandvik Aaseth.

### 3. Checklist – Identification of Inferior Whorl and Image Quality Grading

| **Domain** | **Mosaic continuity and identification of the inferior whorl** | **Completeness of imaging of the inferior whorl region** | **Image quality in the inferior whorl region** |
| --- | --- | --- | --- |
| **Include**  **(1 point)** | Continuous anatomical coverage from the central cornea to the inferior whorl region is present (uninterrupted inferior extension and convergence of sub-basal nerve fibres; seam-like or whorl (spiral) pattern visible).  (Fig. S6a) | Inferior whorl region identifiable despite no more than minor image information loss (e.g. small gaps or reduced contrast).  (Fig. S6b) | Centre (or axis) of the inferior whorl identifiable despite additional structures (e.g. pressure lines, minor motion artefacts, epithelial or stromal cells), with the sub-basal nerve pattern traceable towards the whorl centre.  (Fig. S6c) |
|  | 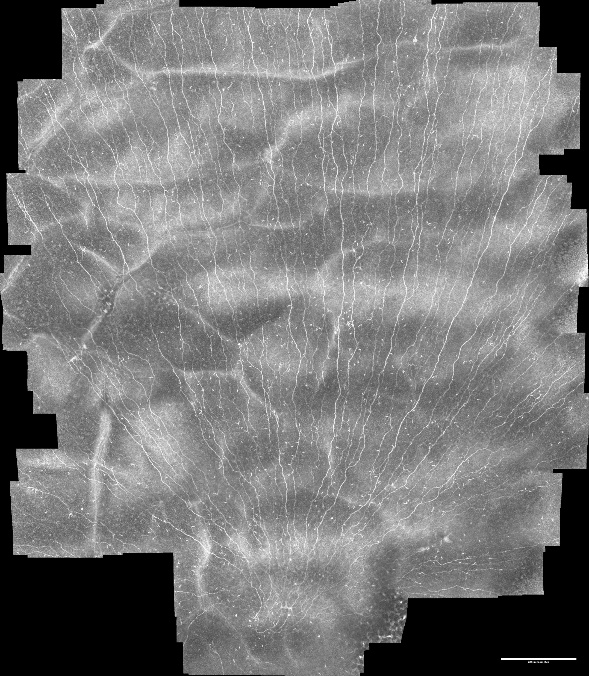 | 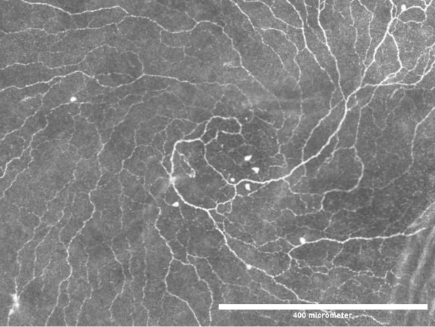 | 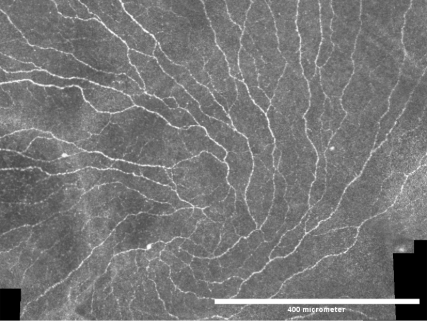 |
|  | **S6a** | ***S6b*** | ***S6c*** |
| **Exclude**  **(0 points)** | Continuous anatomical coverage from the central cornea to the inferior whorl region is absent, preventing identification of the inferior whorl.  (Fig. S6d) | Inferior whorl region unidentifiable due to moderate or severe image information loss (e.g. extensive gaps or poor contrast).  (Fig. 6Se) | Centre (or axis) of the inferior whorl unidentifiable because additional structures obscure the sub-basal nerve pattern.  (Fig. 6Sf) |
|  | 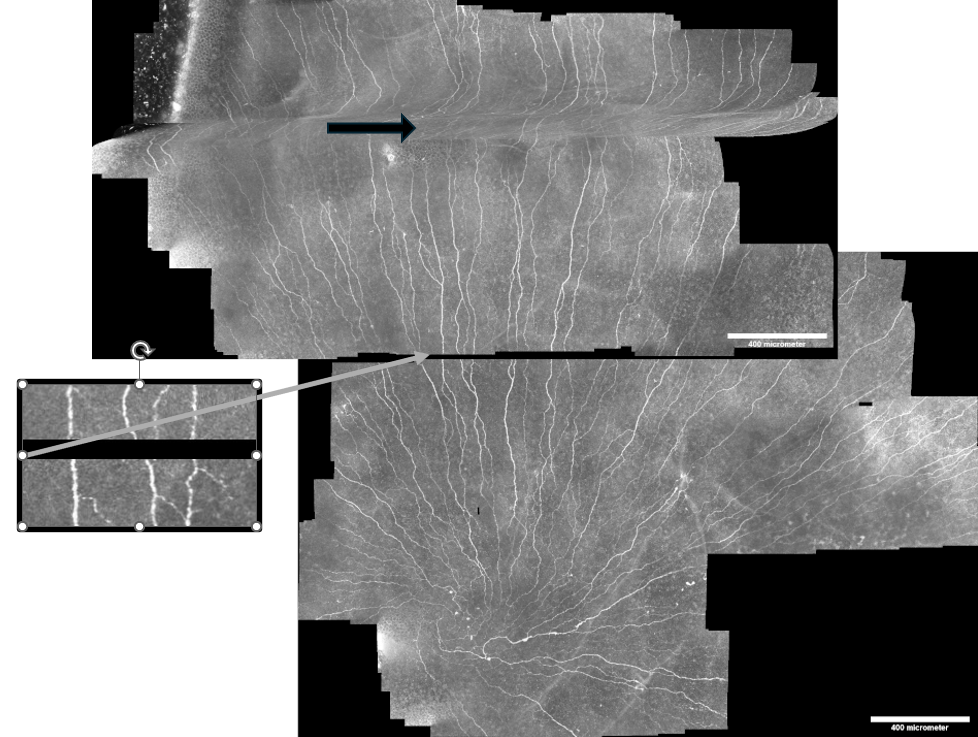 | 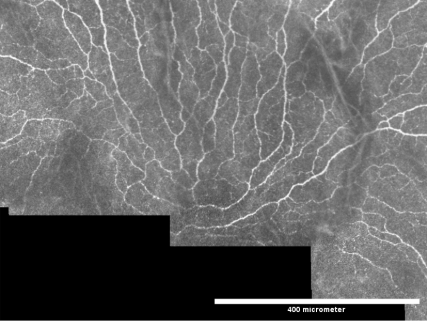 | 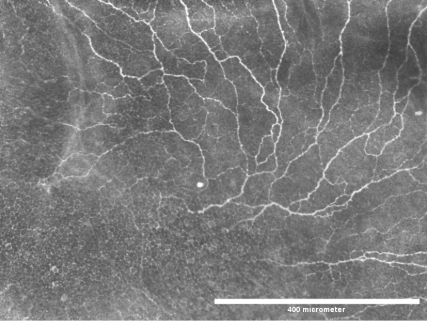 |
|  | ***S6d*** | ***S6e*** | ***S6f*** |

**Figure S6:** The three quality domains with examples of inclusion (**a, b & c**) and exclusion (**d, e & f**). Scoring rule: Total score: 0–3, Include for analysis: Total score = 3, Exclude from analysis: Total score < 3

### 4. Examples of borderline images

| **Borderlines included** | 1. Some missing information inferior of the whorl, but acceptable as the centre of the whorl is still identifiable. 2. Epithelial cells visible, yet the contrast is sufficient for identification of the centre of IW. | 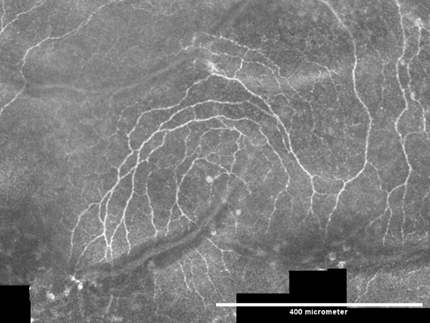 | 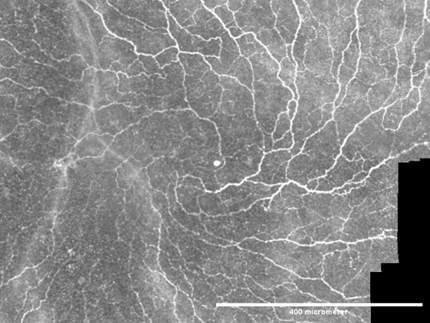 |
| --- | --- | --- | --- |
|  |  | **S7a** | **S7b** |
| **Borderlines excluded** | 1. Although the centre of IW *might be* in the image, the amount of missing information inferior of the IW is too large. 2. Despite good contrast in the majority of the image, other structures are obstructing the visibility of almost all nerve fibres in the bottom left corner. | 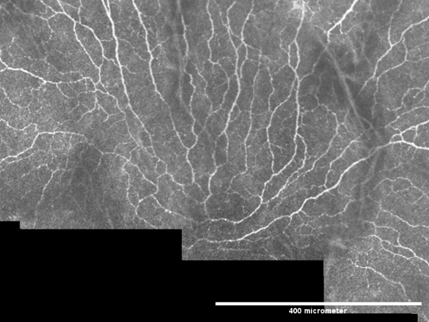 | 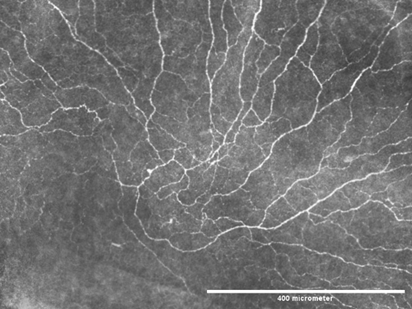 |
|  |  | **S7c** | **S7d** |

**Figure S7**: Examples of borderline images discussed at the consensus meeting. Figures **a** & **b** were included, whereas figures **c** & **d** were excluded

## 3. Defining Centre of Inferior Whorl

The centre of the inferior whorl is the point where the nerve fibres converge to a common end point, with nerve fibres extending upward, downward, and horizontal from this point or an seam-like structure.

When defining the centre, prioritize the following features in order of importance:

1. **Visible common end point**
2. **Overall symmetry**
3. **Presence of nerve fibre bulbs**
4. **Midpoint along the seam**
5. **Orientation of vertical nerve fibres**

### Screen Setup

The magnification described below is based on the screen setup used during centre marking of the inferior whorl:

- **Screen size**: 39.7
- **Resolution**: 2560 x 2160
- **View**: Half of full screen size
- **Scale**: 150%.

Adjustments may be necessary for different setups.

### Step by step process:

1. **Import the mosaic** **image in ImageJ**
   1. File -> Import -> select image for evaluation
2. **Coordinate System**
   1. Top-left corner: **x = 0, y = 0**
   2. x-coordinates increase to the **right**
   3. y-coordinates increase **downwards**
3. **Initial View (33.3% magnification) to localize the inferior whorl region:**
   1. Is there a spiral pattern, seam-like structure or does the nerve fibre converge in a radial pattern?
   2. Look for white end terminals/bulbs to assist localization
4. **Detailed View – Define the centre of the whorl coordinates (75% magnification, Figure S8).**
   1. Left click in the inferior whorl region
   2. + to zoom in (75%)
   3. – to zoom out
   4. Is there a spiral pattern?
   5. Is there a common point where nerve fibres converge? Or is there an seam-like structure?
   6. Do the nerve fibres converge in a clockwise, counterclockwise, towards an seam-like structure or a mixed pattern in the centre of inferior whorl?
   7. Trace fibres from **all** directions to locate the centre of inferior whorl
   8. **If spiral formation:**
      1. Follow fibres to the end point
         - If obvious endpoint: mark with x, y coordinates
         - If no endpoint:
         - Identify symmetrical/geometric centre and mark x, y
   9. **If no spiral:** Is there an seam-like structure?
      1. If yes:
         - Use seam to set y-position
         - Search for vertical fibres coming from central cornea and define the centre in the symmetrical/geometric centre where most vertical nerve fibres meet seam midpoint.
      2. If no seam:
         - Find symmetrical/geometric centre where most vertical nerve fibres converge to a midpoint.
   10. **If both** **spiral and seam**: prioritize spiral steps 4a)
   11. **If undecidable**: mark as *not gradable* (write -1 for x and y)


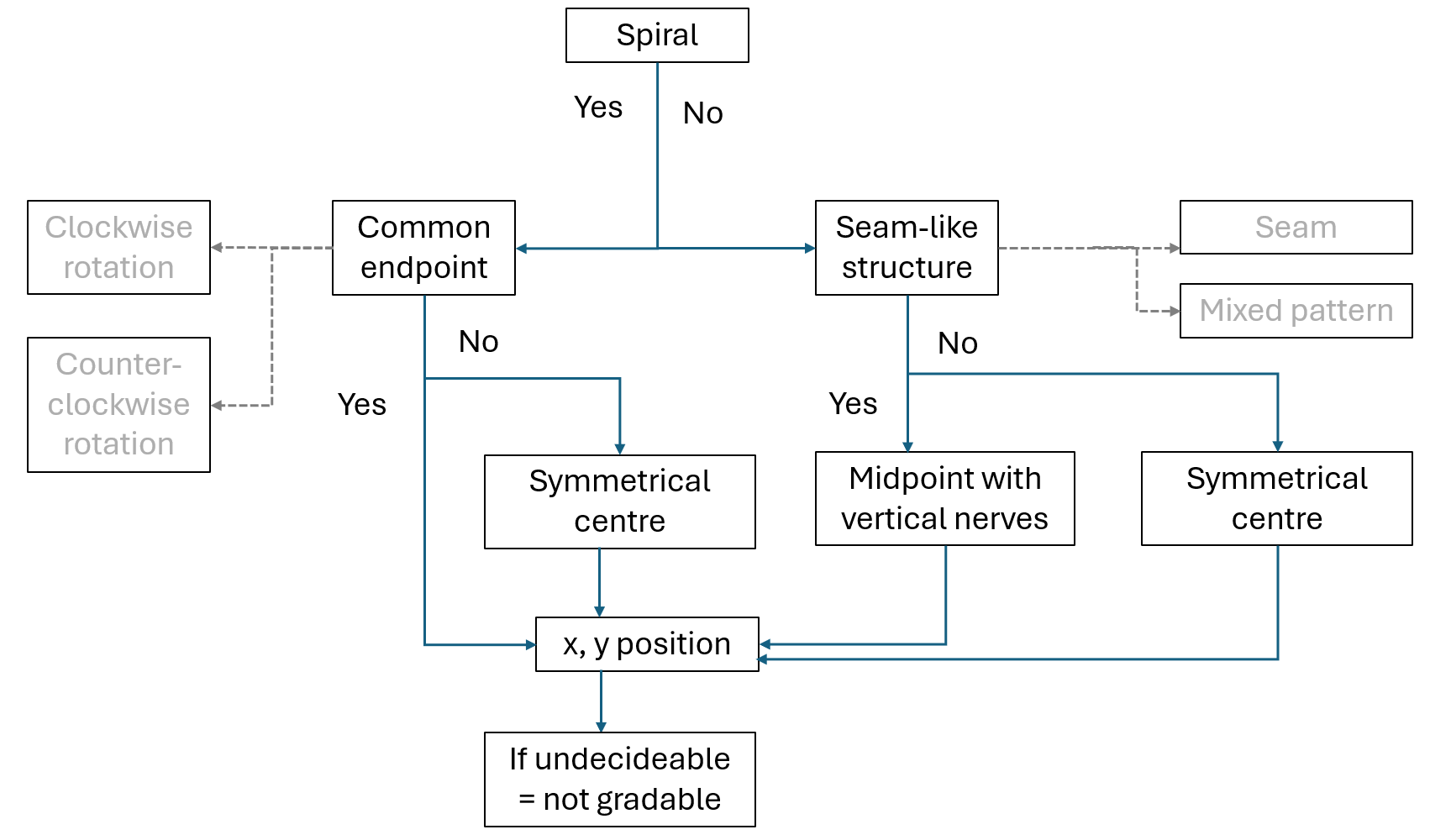


Figure S8: Flow diagram for setting the x, and y coordinates for the centre of inferior whorl. Dashed grey lines and grey words is to remember to note the rotation of the whorl.

### References

1. Heidelberg Engineering. Heyex 2 and Heyex Pacs User Manual. 2021;

2. Heidelberg Engineering. HRT3 Rostock Cornea Module Hardware Manual. 2021;

3. Käser E. Fixation_for_IVCM [Internet]. 2025 [cited 2025 Feb 12];Available from: https://github.com/emakaeser/Fixation_for_IVCM

4. Sandvik SA. pattern_whorl5x4_15x16.txt [Internet]. 2025 [cited 2025 Sept 12];3094450 Bytes. Available from: https://usn.figshare.com/articles/dataset/pattern_whorl5x4_15x16_txt/30102349/1
