# Supplementary material for: A novel method for standardised imaging of corneal subbasal nerves by in vivo confocal microscopy – a pilot validation study
Source: Sci Rep. 2026 Jun 9;16:22682. doi: 10.1038/s41598-026-54268-8 (PMC13385856; doi:10.1038/s41598-026-54268-8)
Supplement: Supplementary file 18 — Supplementary Material 18 [file 41598_2026_54268_MOESM18_ESM.pdf]

Assessment of Personal Data Processing

Reference number: 249925
Type of assessment: Standard
Date: 15 May 2024

Project Title

Pilot study of a method for imaging the corneal subbasal nerve plexus using *in vivo* confocal microscopy

---

Data Controller Institution

University of South-Eastern Norway
Faculty of Health and Social Sciences
Department of Optometry, Radiography and Lighting Design

Project Manager

Vibeke Sundling

Academic Level

Research / PhD project

Project Period

15 April 2024 – 30 September 2026

Categories of Personal Data

General personal data

- Name
- Date of birth
- Contact information

Special categories of personal data

- Health information

Legal Basis

Consent, in accordance with GDPR Article 6(1)(a)

USN
Rett kopi/Certified copy

Description of the Processing

Research purpose and justified need for personal data

The purpose of the study is to evaluate a method for imaging the corneal subbasal nerve plexus using *in vivo* confocal microscopy.

Intra- and inter-repeatability of imaging a defined area of the cornea will be assessed based on imaging performed using a standardized method and procedure by two optometrists.

Furthermore, the study will explore different methods for analyzing corneal nerve fibers in the subbasal plexus.

Personal data such as name and date of birth will only be used in connection with data collection and will not be processed further.

Personal data will not be used for additional purposes.

Number of Participants

1–99

Sample Group

Healthy individuals aged 20–30 years

Recruitment is carried out through the institution's network, via open invitations to employees (email) and students (announcements on the learning platform) at the Department of Optometry, Radiography and Lighting Design.

Age Group

20–30 years

Data Collection / Method

- Other

During recruitment, individuals aged 20–30 years without known disease are invited to participate.

Personal data are collected at the start of data collection.

Participants who consent will have images taken of the corneal subbasal nerve plexus by two optometrists.

It is not possible to identify participants based on the images obtained.

Rett kopi/Certified copy

Overall Assessment

We have assessed that the processing of personal data complies with the requirements of the data protection regulations. These assessments are carried out on behalf of the data controller institution.

You must follow the institution's guidelines for information security, as well as any conditions outlined in this assessment.

The Personal Data Services unit has assessed the changes registered in the notification form.

It is our assessment that the processing of personal data in the project will comply with data protection legislation, provided that it is conducted in accordance with what is documented in the notification form and its attachments.

The project may proceed.

Follow-up of the Project

We will follow up at the planned project completion to clarify whether the processing of personal data has ended.

For long-term projects, we will make contact every two years to remind you that any changes must be reported.

Best of luck with your project!

USN

Rett kopi/Certified copy
