# Supplementary material for: A novel method for standardised imaging of corneal subbasal nerves by in vivo confocal microscopy – a pilot validation study
Source: Sci Rep. 2026 Jun 9;16:22682. doi: 10.1038/s41598-026-54268-8 (PMC13385856; doi:10.1038/s41598-026-54268-8)
Supplement: Supplementary file 19 — Supplementary Material 19 [file 41598_2026_54268_MOESM19_ESM.pdf]

Would you like to participate in the research project
Imaging and analysis of the corneal nerves?

Purpose of the project
This is a question for you if you would like to participate in a research project where the aim is to validate a method for imaging of the corneal nerve fibres. The project is part of a larger PhD work with an overall aim to develop a method for imaging of the corneal nerve fibres for clinical practice and clinical research.

Why are you being asked to participate?
You receive this request because we want to examine healthy individuals without known diseases or eye conditions, who do not use contact lenses or have binocular vision problems. The invitation is sent to all students at the University of South-Eastern Norway. The research project is conducted at the National Centre for Optics, Vision, and Eye Care's research.

Which institution is responsible for the research project?
Institute for Optometry, Radiography, and Lighting Design, Faculty of Health- and Social science at the University of South-Eastern Norway is responsible for the personal data processed in the project.

Participation is voluntary
Participation is voluntary, and it will not have any negative consequences if you decide not to participate or want to withdraw your consent at later stages.

What does participation involve for you?
By participation there is necessary to meet for an examination where your eyes are examined over one or two visits dependent if you have the possibility to participate once or twice. Two persons will take images of your cornea with the use of an in vivo confocal microscopy and each imaging series takes approximately 5-10 minutes. In total, three series of images on one eye will be conducted the first time. The examination takes 30-45 minutes for one eye. Please schedule 1h in total of your time for each visit.

There is no risk connected to participation. The confocal microscope uses an helium neon diode laser with the wavelength of 670 nm, which is class I laser and makes no harm to the eye. Local anesthetic will be used (0.5% Oxibuprocaine) as standard procedure due to the contact between the microscope and the eye. These eye drops may sting a little. In addition, there will be used a contact gel (Viscotears) to facilitate for good contact between the cornea and the microscope. The ocular surface will be examined both in advance and after the examination with the use of fluorescein. This is a contrast color which will be washed away by your tears. Some do experience some feeling of dryness and transient blurred

vision. However, you will get artificially tears, eyedrops, to use if you experience dryness after the examination. In addition, we will take topography measurements of your eyes to explore if there are any structural conditions that affect the imaging.

Your personal privacy – how we will store and use your personal data
We will register your name, date of birth, and contact details. All personal data will be recorded with an ID-code. The results from the imaging will be registered with this ID. To facilitate for privacy the connection key between the personal data and the ID will be stored safely and deleted by the end of the project, no later thatn the 30th of September 2026. All data that are stored will be anonymized.

We will only use the information for the aim stated in this letter. We will treat your personal data in accordance with the privacy regulations. You can read more about privacy below.

Best regards

Project manager
Professor Vibeke Sundling
Supervisor

Project employee
Siv Aaseth Sandvik and Eilin Lundanes
PhD students

USN
Rett kopi/Certified copy
24.04.26 [illegible]

In depth information about privacy – how we store and use your data

Participation is voluntary and you can withdraw your consent.

It is voluntary to participate in this project. If you want to participate, you sign the consent form on the last page. You can withdraw your consent at any time without giving a reason. It will not have any consequences for you. If you withdraw your consent, there will be no further research on your information. If desired, you can request access to the information stored about you, and it will be provided within 30 days. Please contact one of the research staff. You can also request your information to be deleted. Deletion does not apply if the information or material has been de-identified, analyzed or published. Publications will not contain personal data or identifiable information.

Contact project manager Professor Vibeke Sundling (vibeke.sundling@usn.no) or one of the PhD students Siv Aaseth Sandvik (siv.a.sandvik@usn.no) or Eilin Lundanes (eilin.lundanes@usn.no) if you want to withdraw your consent or have any questions regarding the research project. Those are the only staff members that have access to your personal data.

What happens with your personal data?

All information will be processed without names and personal ID or other directly identifying details (=coded information). A code links you to your information through a name list. Data will be stored on a secure research server accessible only by the project manager and research staff, Phd students. De-identified images will be exported and transferred by secure cloud-based service at the Karlsruhe Institute for Technology, which is jointly responsible for data processing. Your personal information will not be accessible to collaborating data processors and will be deleted at the end of the project.

Your rights

As long as you can be securely identified in the data set, you have the right to object, request access, correction and/or deletion of your personal data. If you contact us about your rights, we will reply within a month. We will give you a good reason if we believe that you cannot be securely identified, or that the rights cannot be exercised. You also have the right to complain to the Norwegian Data Protection Authority about how we process your information.

Project manager, Professor Vibeke Sundling, Institute for Optometry, Radiography, and Lighting Design, Faculty for Health and Social Science, University of South-Eastern Norway and National Centre for Optics, Vision and Eye Care has the main responsibility for the research project and that your personal data will be stored securely. Project manager can be contacted by mobile phone +47 924 24 360, or Vibeke.sundling@usn.no

What gives us the right to process personal data about you?

We process information about you for purposes related to scientific research, and because the research project is considered to be in the public interest. We have taken measures that ensures that the privacy of the data subjects is protected and treat your personal data based on your consent.

On behalf of, Institute for Optometry, Radiography, and Lighting Design, Faculty for Health and Social Science, University of South-Eastern Norway, the Data Protection Services at Sikt - Norwegian Agency for Shared Services in Education and Research, have assessed that the processing of personal data in this project is in accordance with the privacy regulations.

What happens with your personal data after the end of the research project?
This research project is planned to end by the 30th of September 2026. Personal data will then be deleted.

Questions
If you have questions or want to exercise your rights, please contact:

- Professor and supervisor Vibeke Sundling (vibeke.sundling@usn.no
- PhD student Siv Aaseth Sandvik (siv.a.sandvik@usn.no)
- PhD student Eilin Lundanes (eilin.lundanes@usn.no)
- Our Data Protection Officer: Pål A. Solberg (paal.a.solberg@usn.no)

If you have questions related to Sikt's assessment of the project, you can contact by email: personverntjenester@sikt.no, or by phone: + 47 73 98 40 40.

I GIVE CONSENT FOR MY PERSONAL DATA TO BE USED AND PROCESSED AS DESCRIBED UNTIL THE END OF THE PROJECT

I PARTICIPATE VOLUNTEERLY

Place and date

Signature of the participant

Name of the participant with capital letters

I confirm to have given information about the research project and the participant have got the possibility to ask questions.

Place and date

Signature

USN
Rett kopi/Certified copy
24.04.26 Marle Bones
